# Supplementary figures and images for: Rho GTPase signaling and mDia facilitate endocytosis via presynaptic actin
Source: eLife. 2024 Mar 19;12:RP92755. doi: 10.7554/eLife.92755 (PMC10950329; doi:10.7554/eLife.92755)

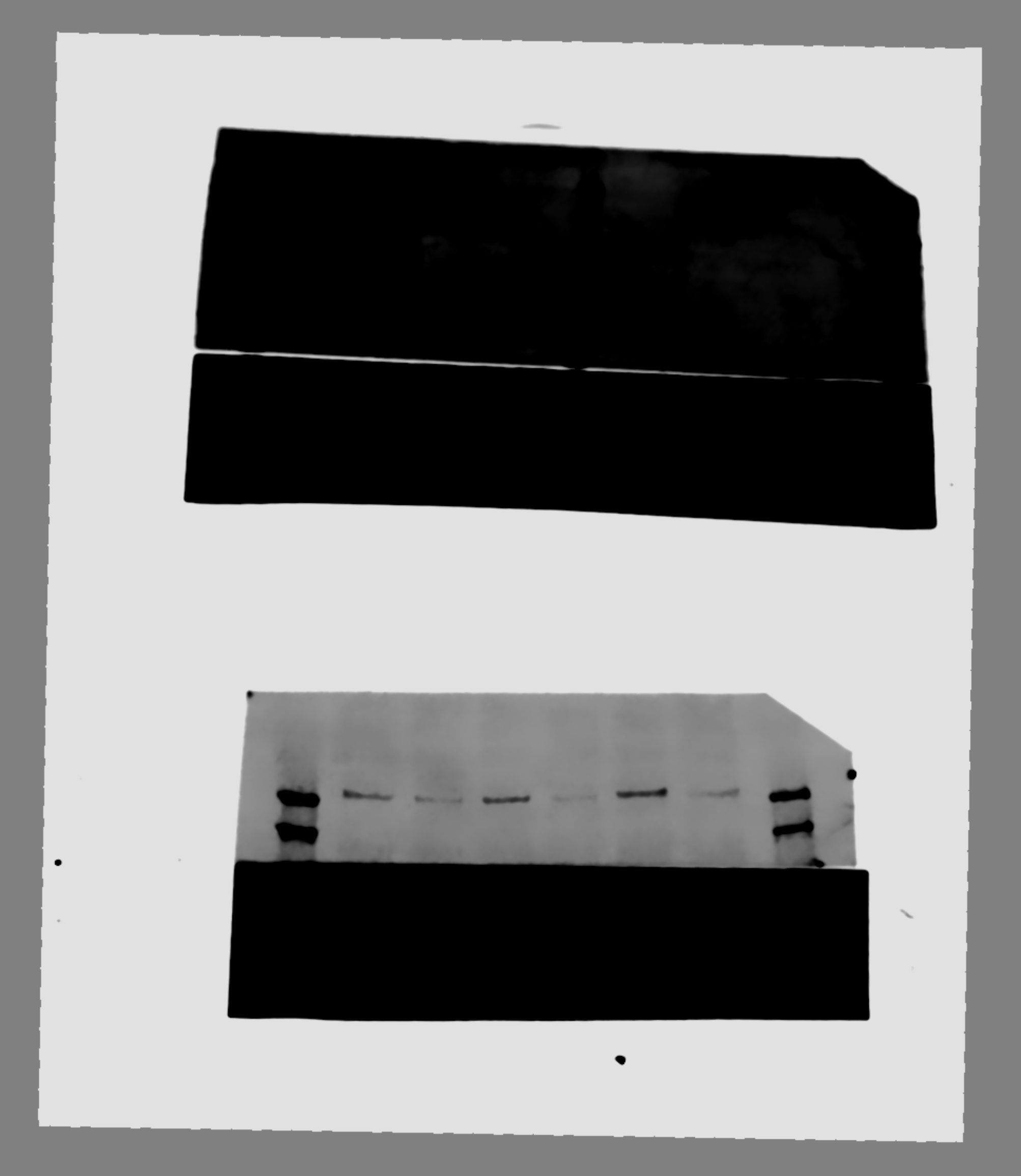

Supplement: Figure 1—figure supplement 1—source data 2. [file elife-92755-fig1-figsupp1-data2.zip › Figure 1-Figure Supplement 1-Source Data 2.tif]

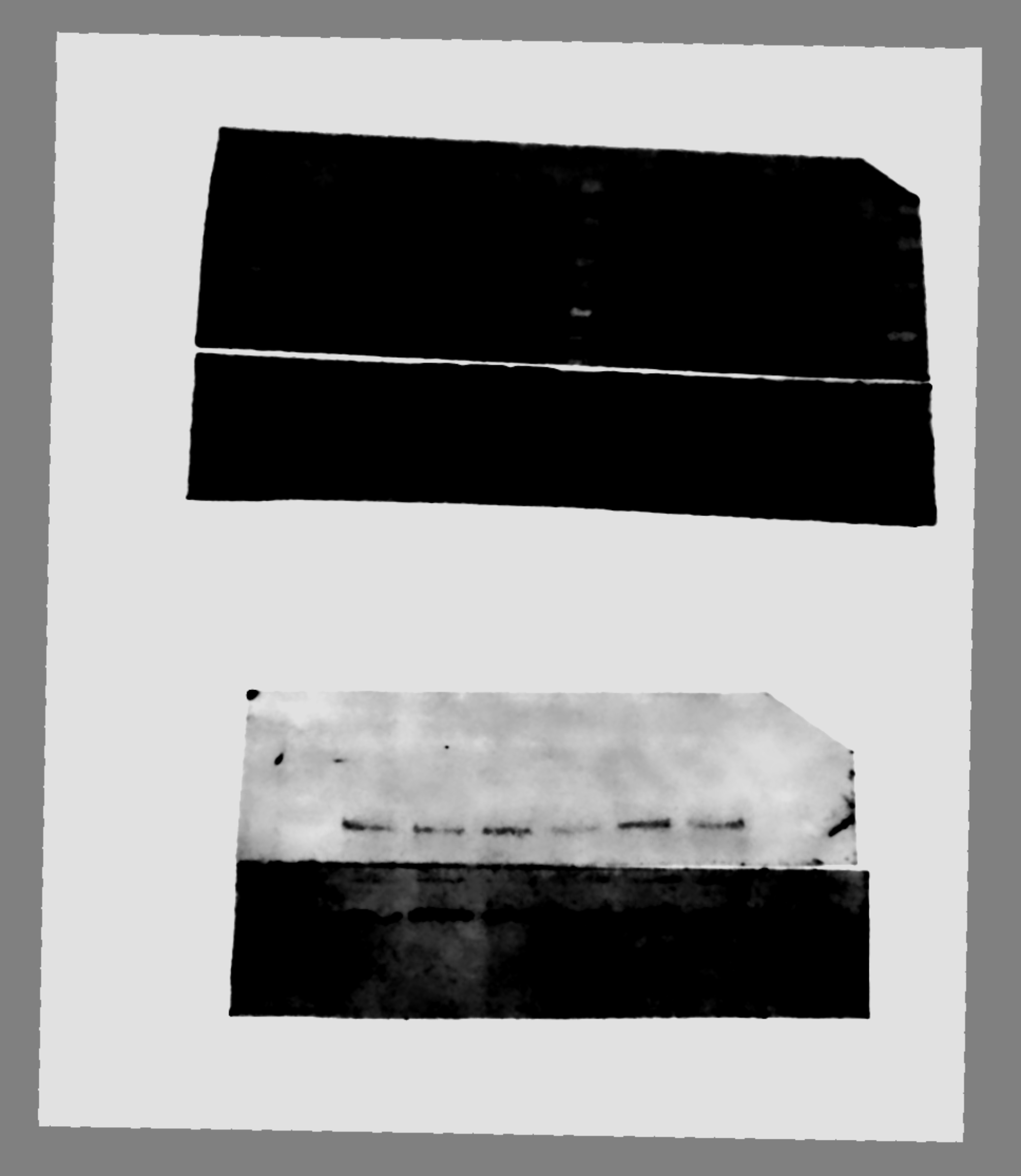

Supplement: Figure 1—figure supplement 1—source data 3. [file elife-92755-fig1-figsupp1-data3.zip › Figure 1-Figure Supplement 1-Source Data 3.tif]

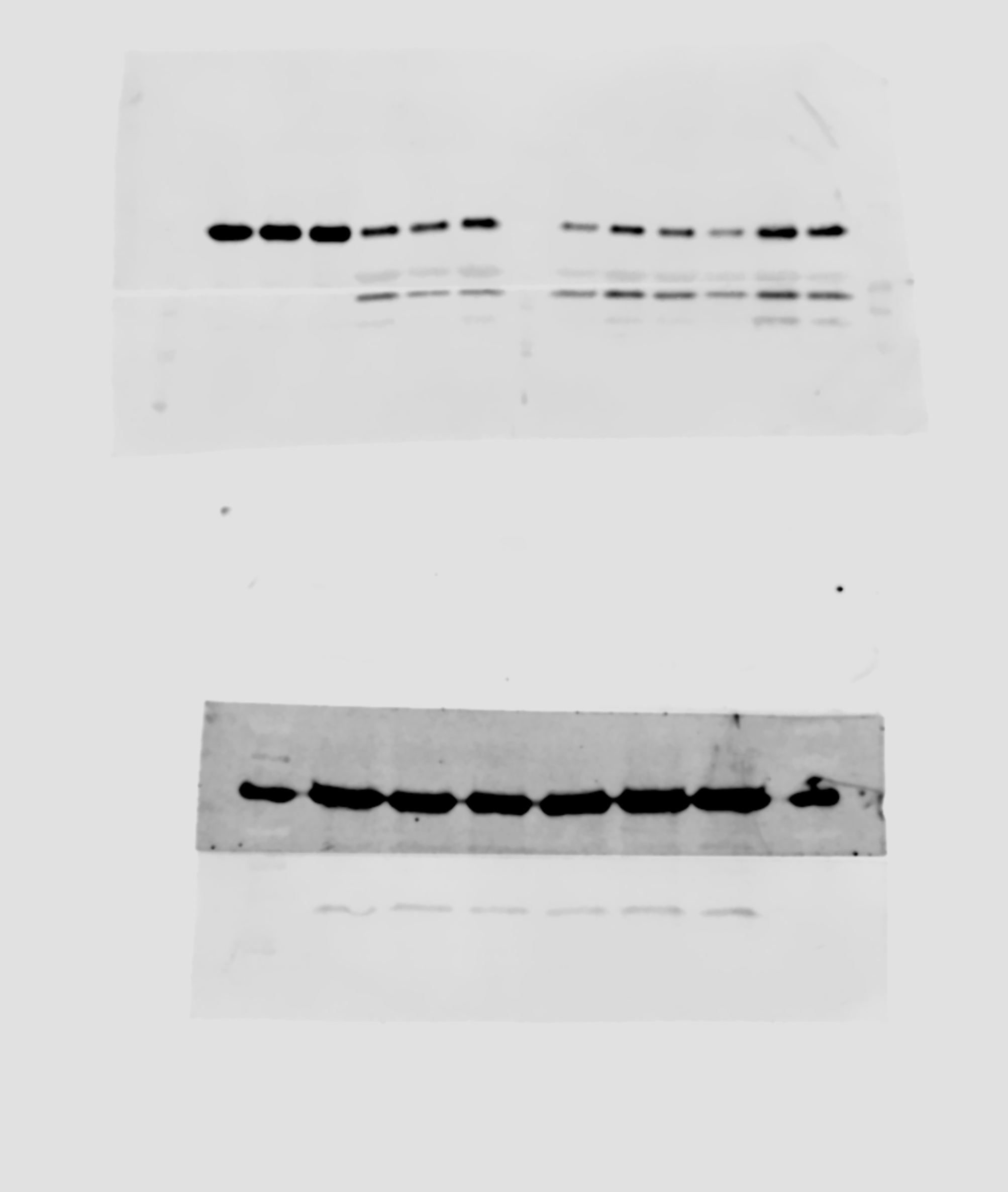

Supplement: Figure 1—figure supplement 1—source data 4. [file elife-92755-fig1-figsupp1-data4.zip › Figure 1-Figure Supplement 1-Source Data 4.tif]

Figure 1 S1

A

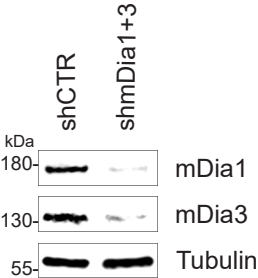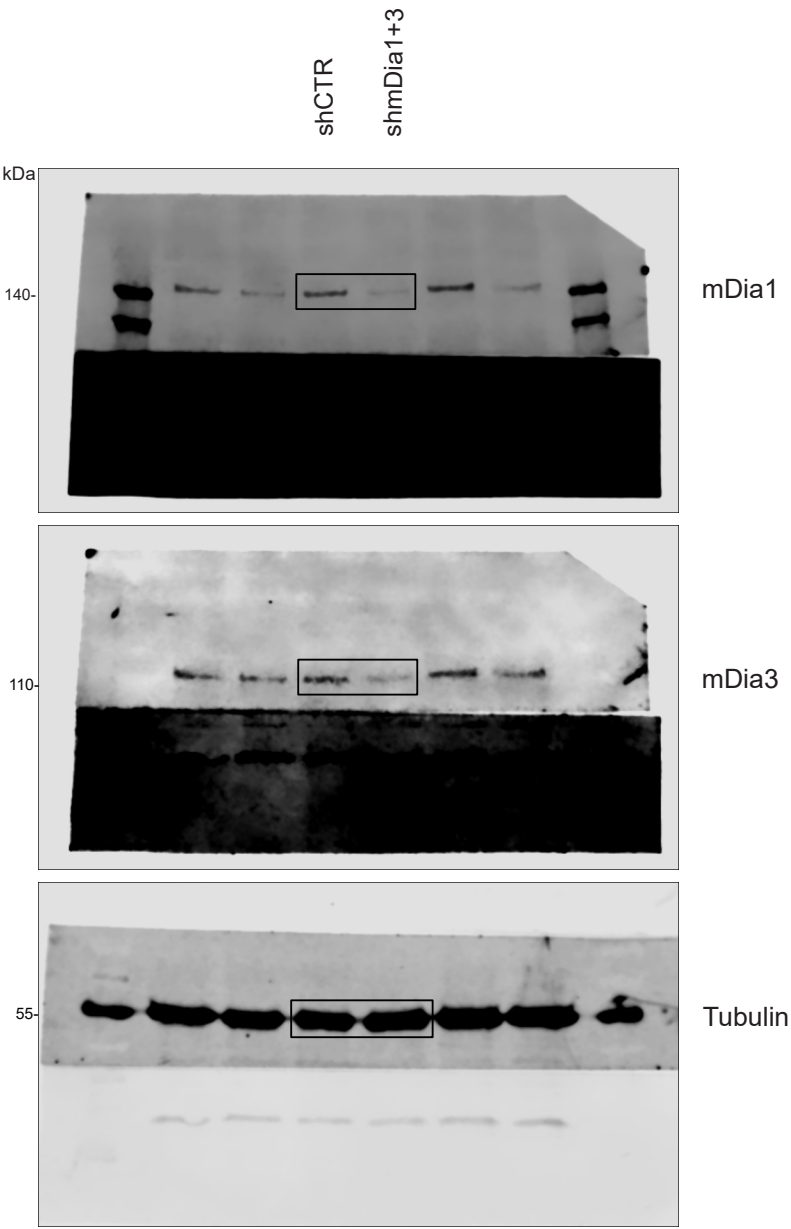

Supplement: Figure 1—figure supplement 1—source data 5. [file elife-92755-fig1-figsupp1-data5.zip › Figure 1-Figure Supplement 1-Source Data 5.pdf]

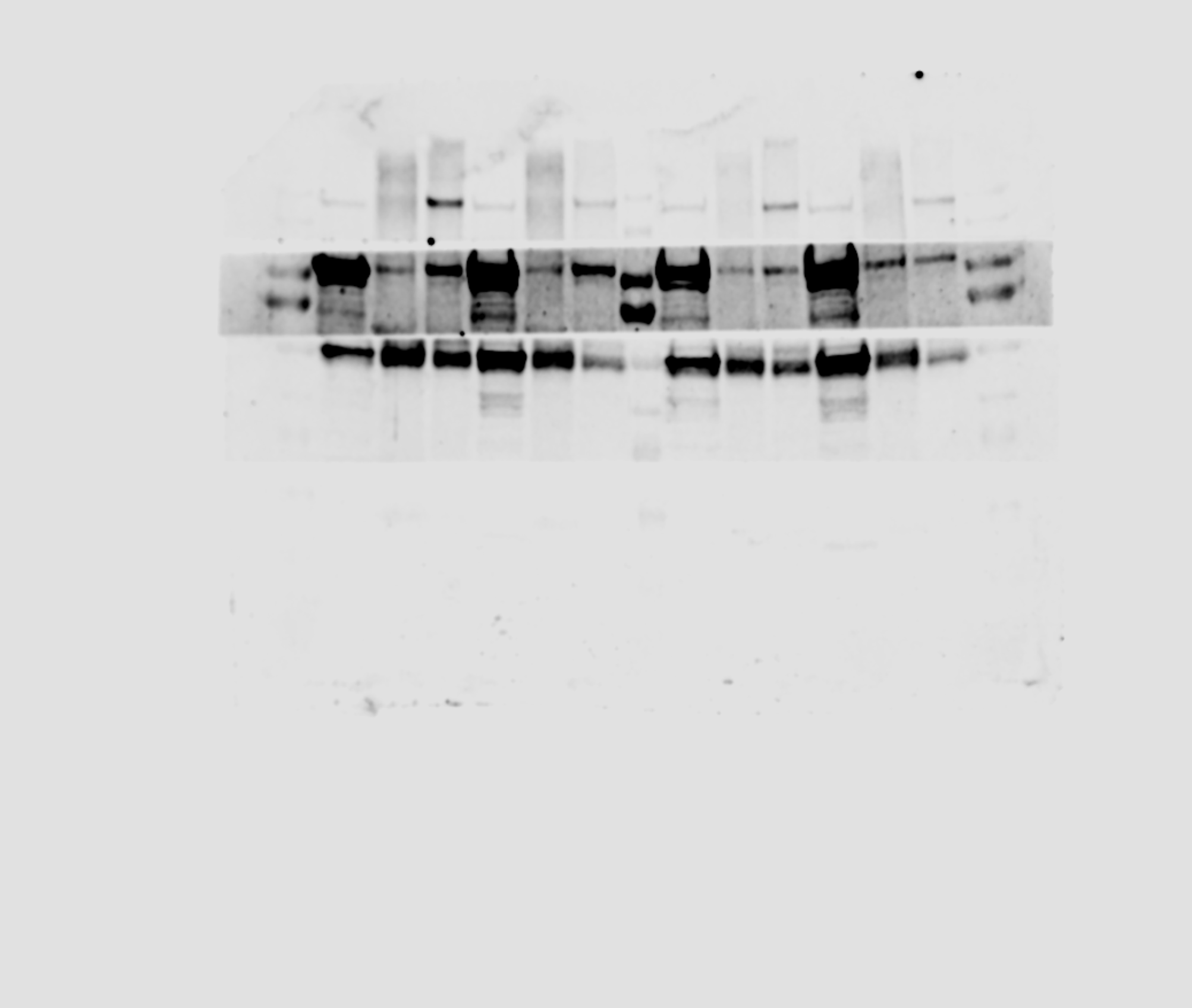

Supplement: Figure 3—source data 2. [file elife-92755-fig3-data2.zip › Figure 3-Source Data 2.tif]

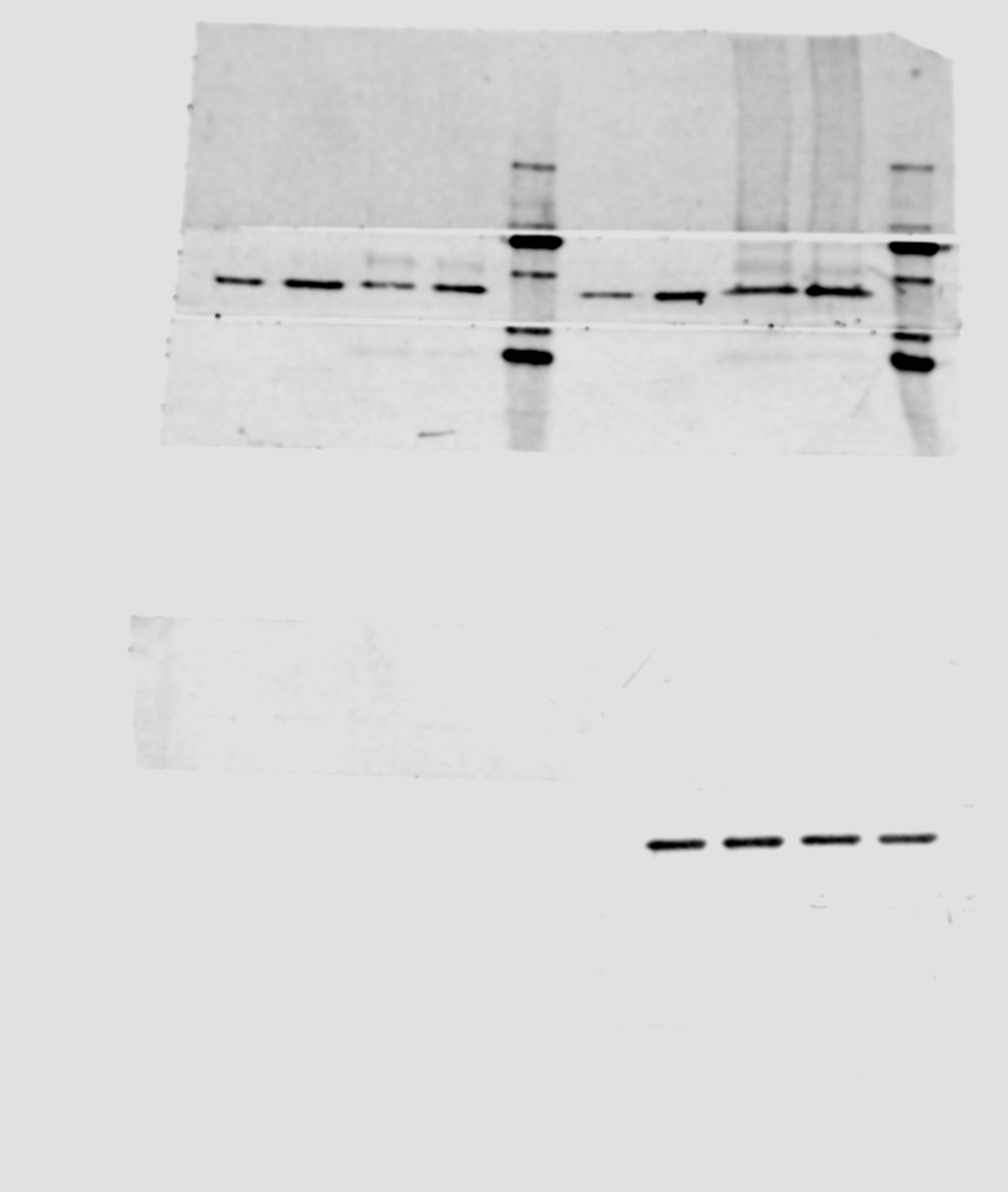

Supplement: Figure 3—source data 3. [file elife-92755-fig3-data3.zip › Figure 3-Source Data 3.tif]

Figure 3

F

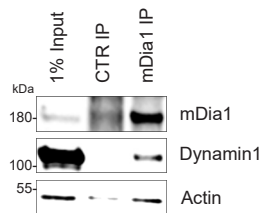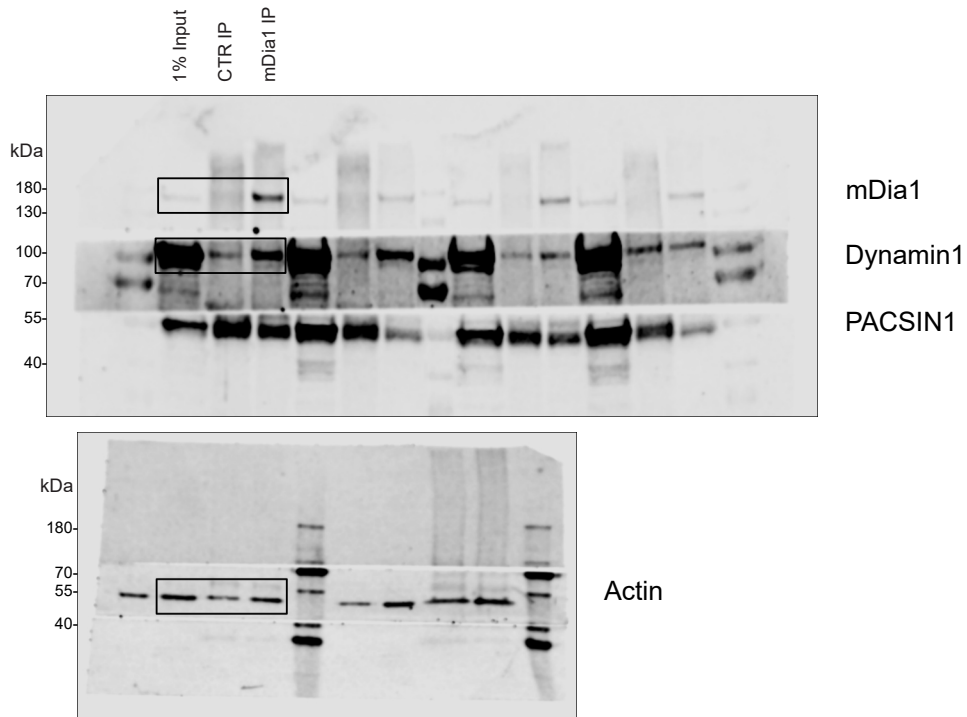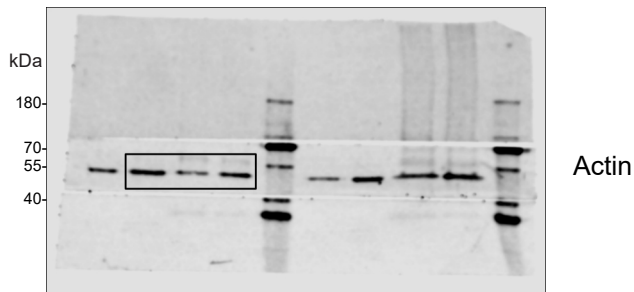

Supplement: Figure 3—source data 4. [file elife-92755-fig3-data4.zip › Figure 3-Source Data 4.pdf]

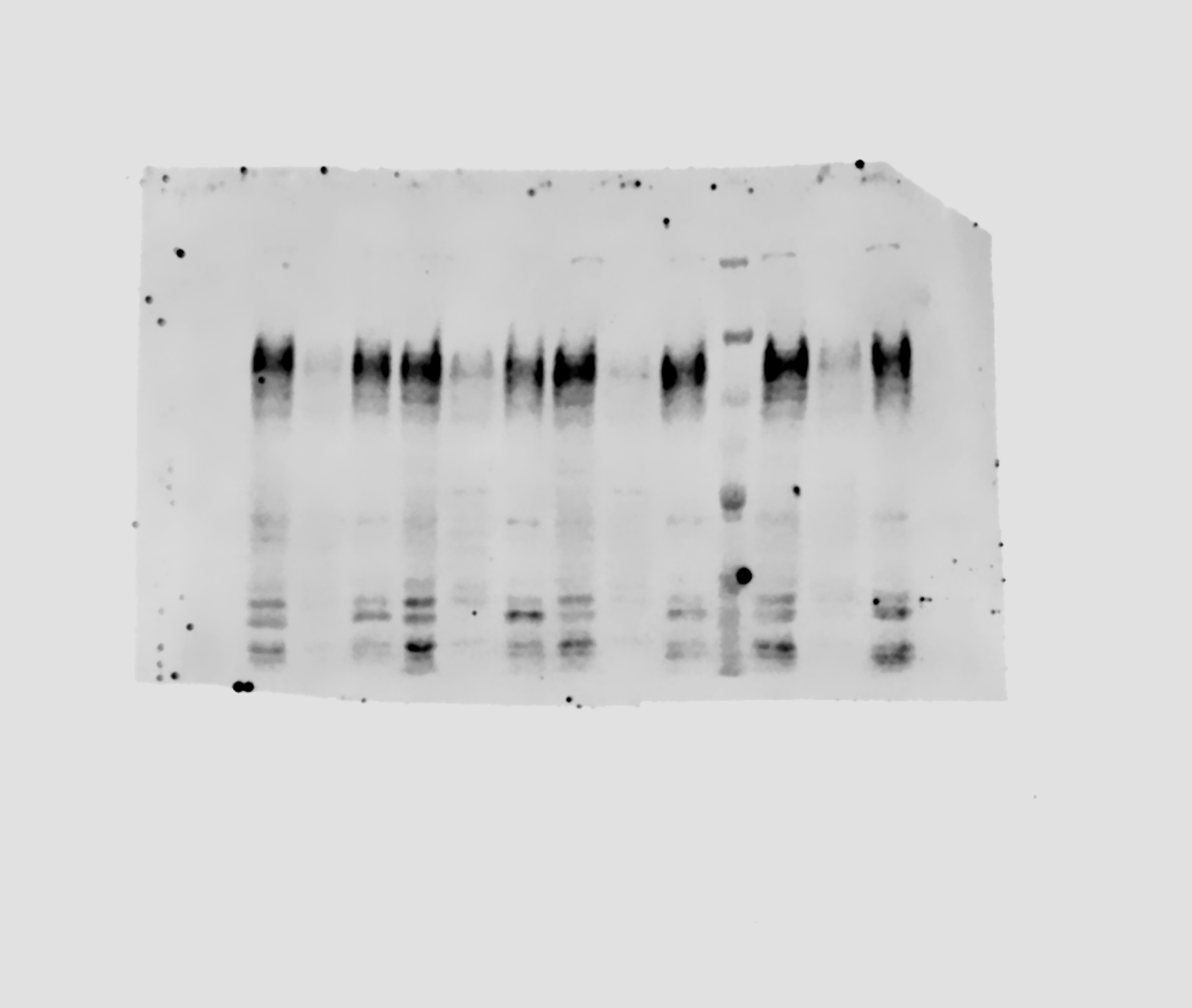

Supplement: Figure 3—figure supplement 1—source data 2. [file elife-92755-fig3-figsupp1-data2.zip › Figure 3-Figure Supplement 1-Source Data 2.tif]

Figure 3 S1

A

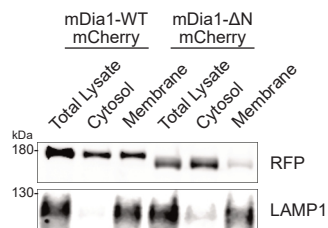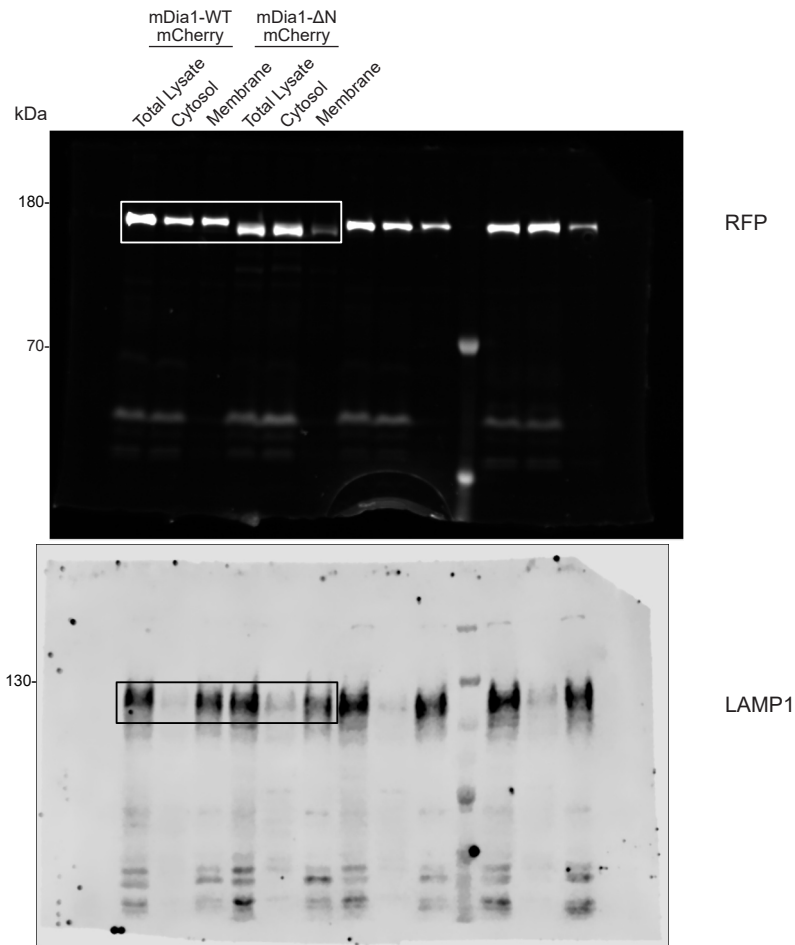

Supplement: Figure 3—figure supplement 1—source data 3. [file elife-92755-fig3-figsupp1-data3.zip › Figure 3-Figure Supplement 1-Source Data 3.pdf]

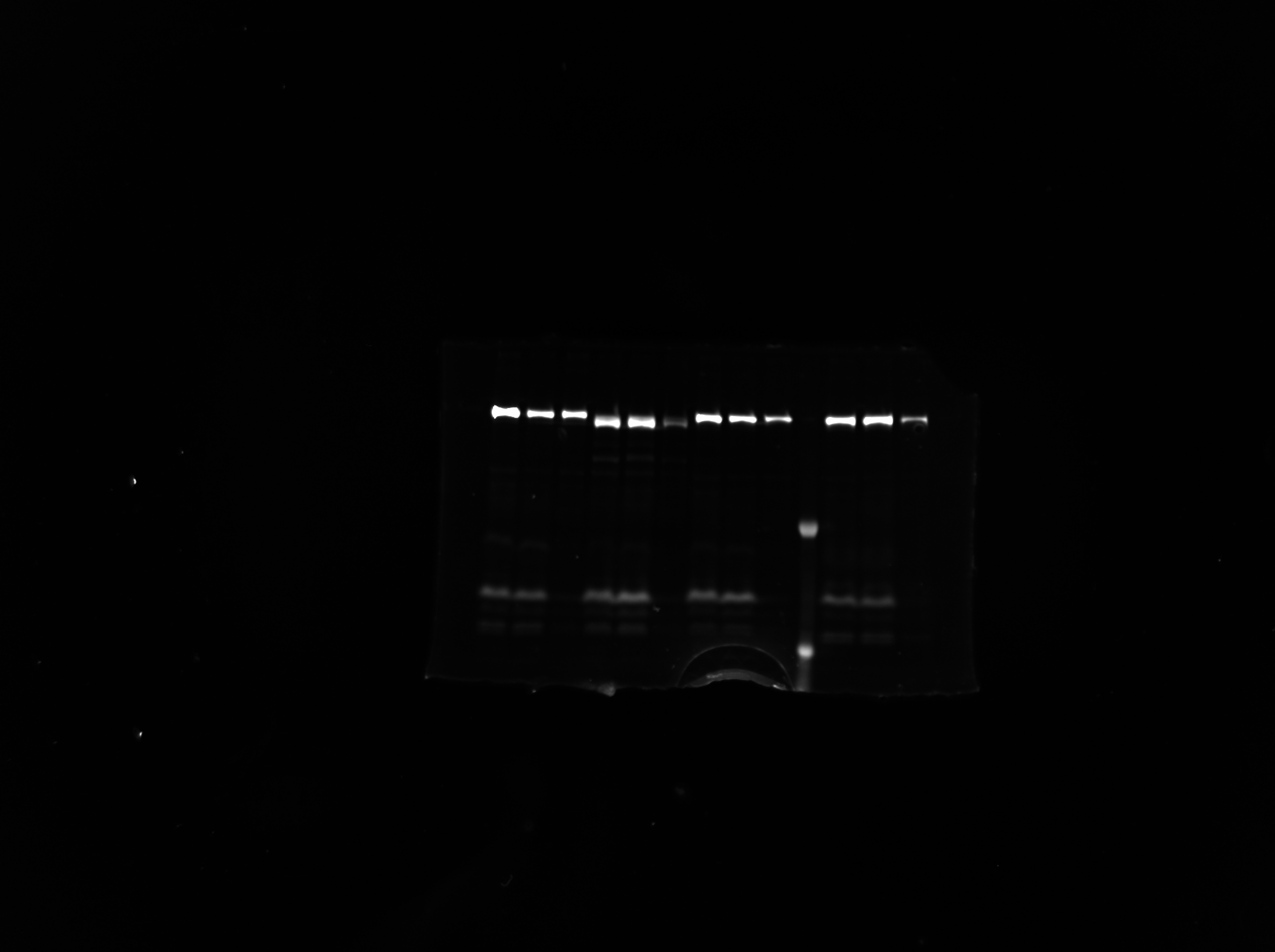

Supplement: Figure 3—figure supplement 1—source data 5. [file elife-92755-fig3-figsupp1-data5.zip › Figure 3-Figure Supplement 1-Source Data 5A.tif]

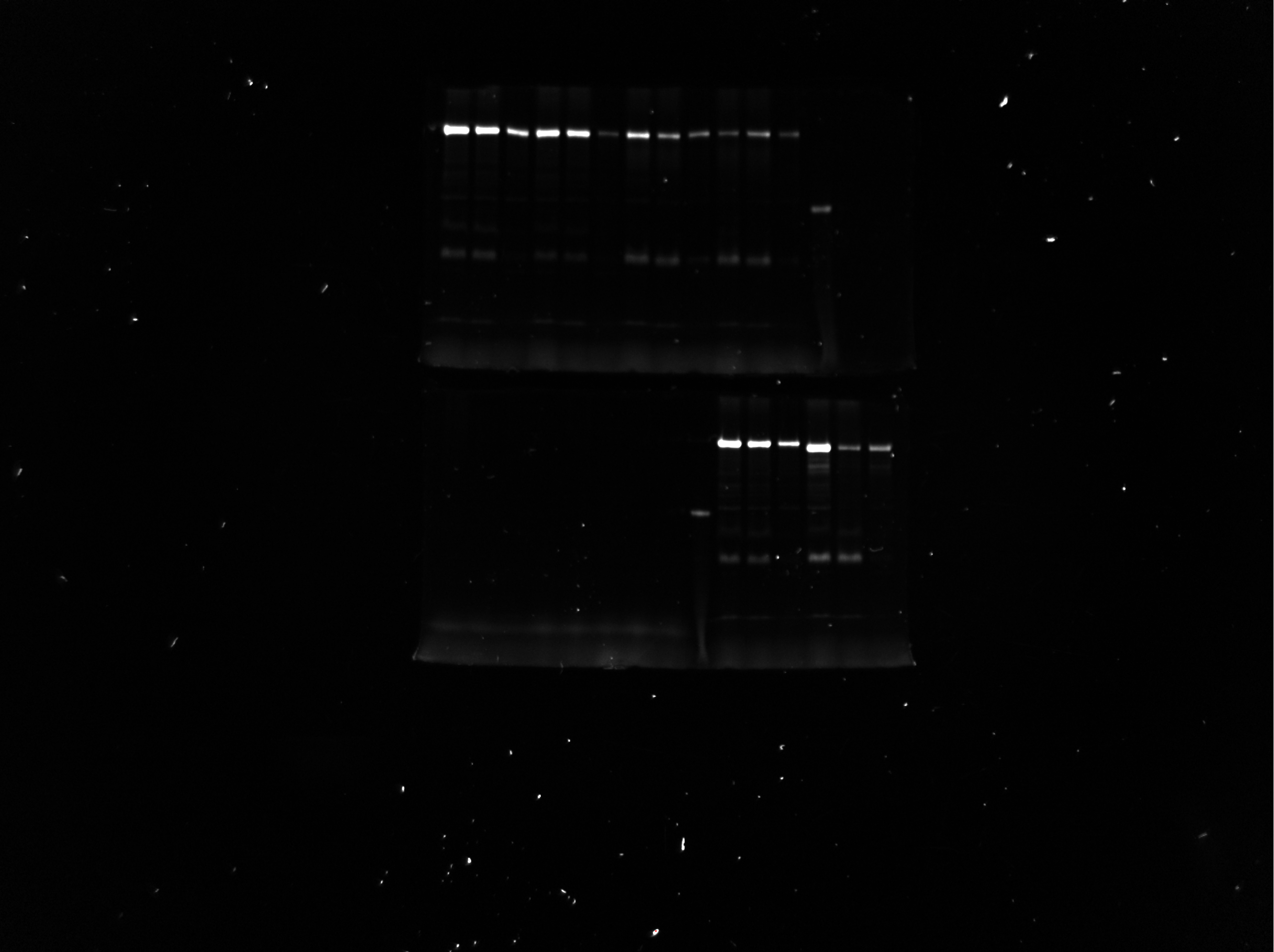

Supplement: Figure 3—figure supplement 1—source data 5. [file elife-92755-fig3-figsupp1-data5.zip › Figure 3-Figure Supplement 1-Source Data 5B.tif]

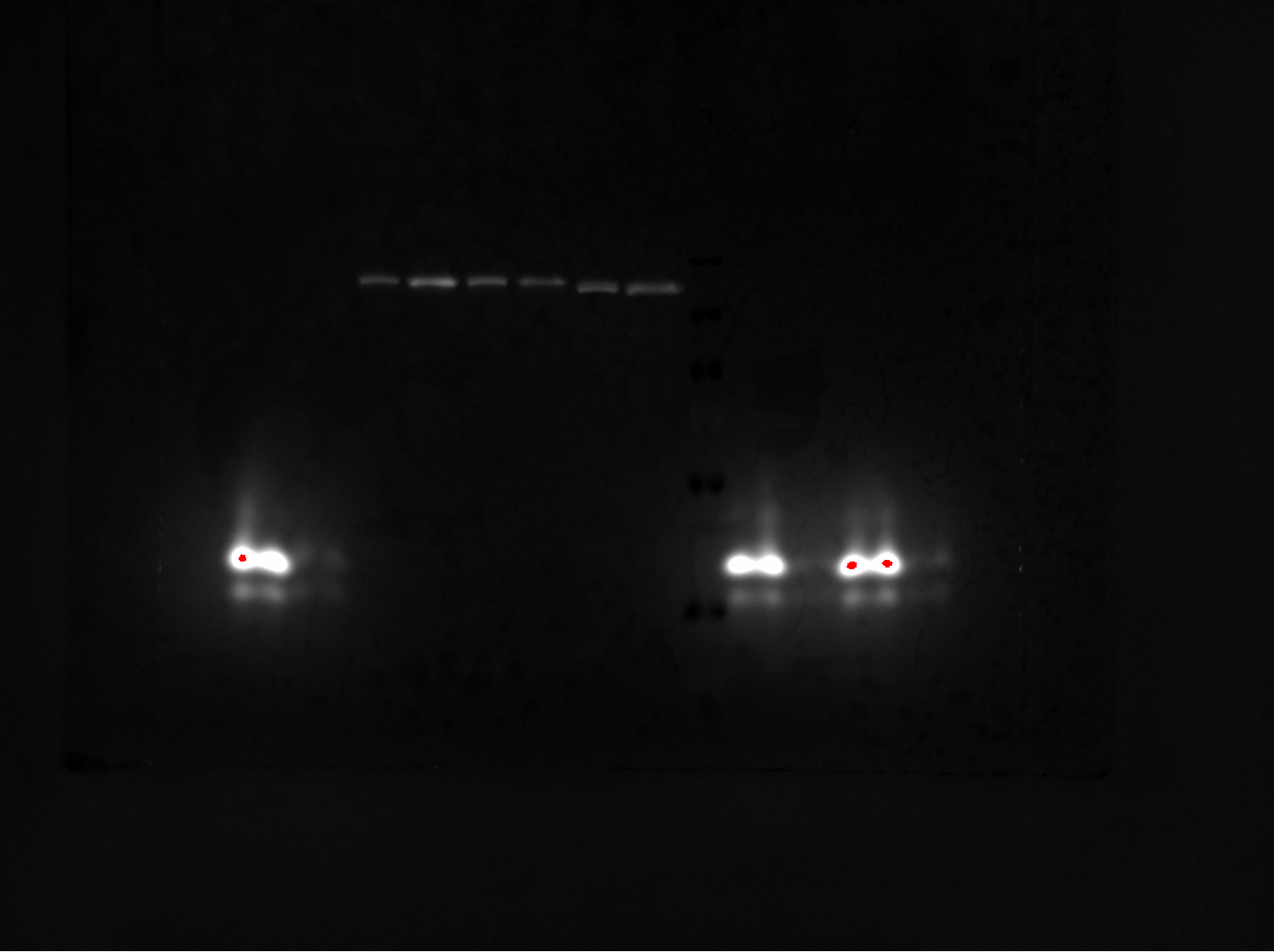

Supplement: Figure 3—figure supplement 1—source data 5. [file elife-92755-fig3-figsupp1-data5.zip › Figure 3-Figure Supplement 1-Source Data 5C.tif]

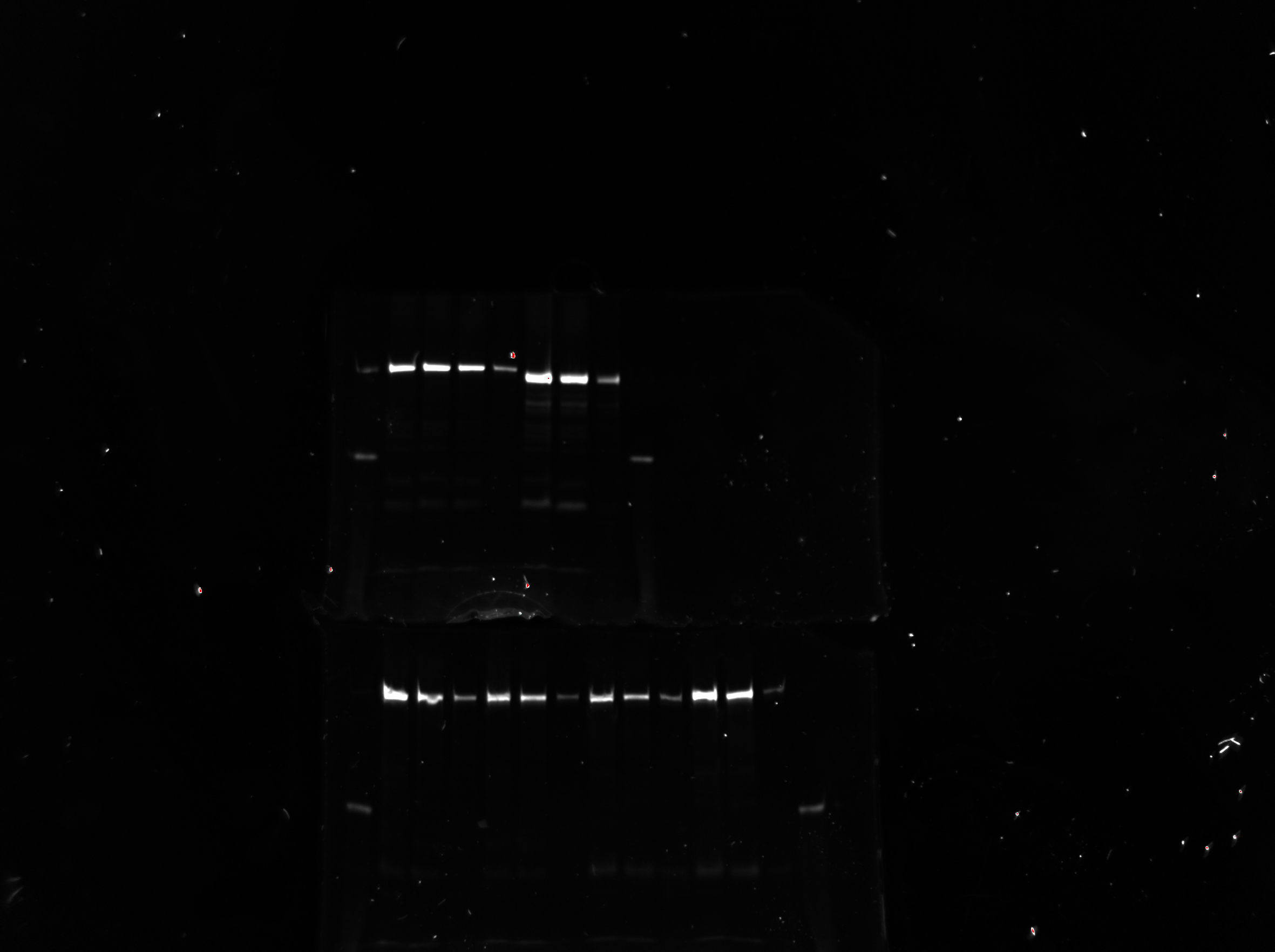

Supplement: Figure 3—figure supplement 1—source data 5. [file elife-92755-fig3-figsupp1-data5.zip › Figure 3-Figure Supplement 1-Source Data 5D.tif]

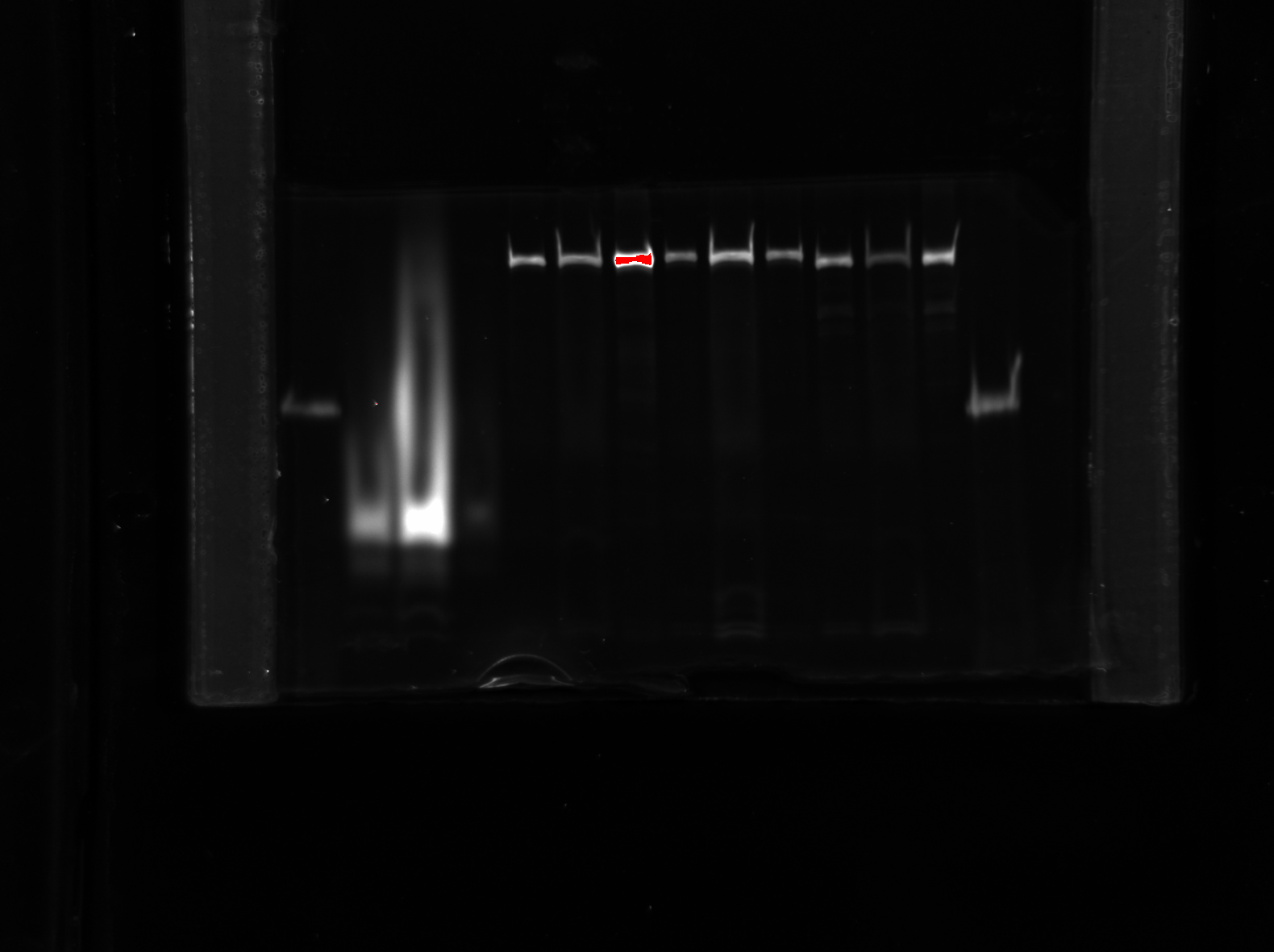

Supplement: Figure 3—figure supplement 1—source data 5. [file elife-92755-fig3-figsupp1-data5.zip › Figure 3-Figure Supplement 1-Source Data 5E.tif]

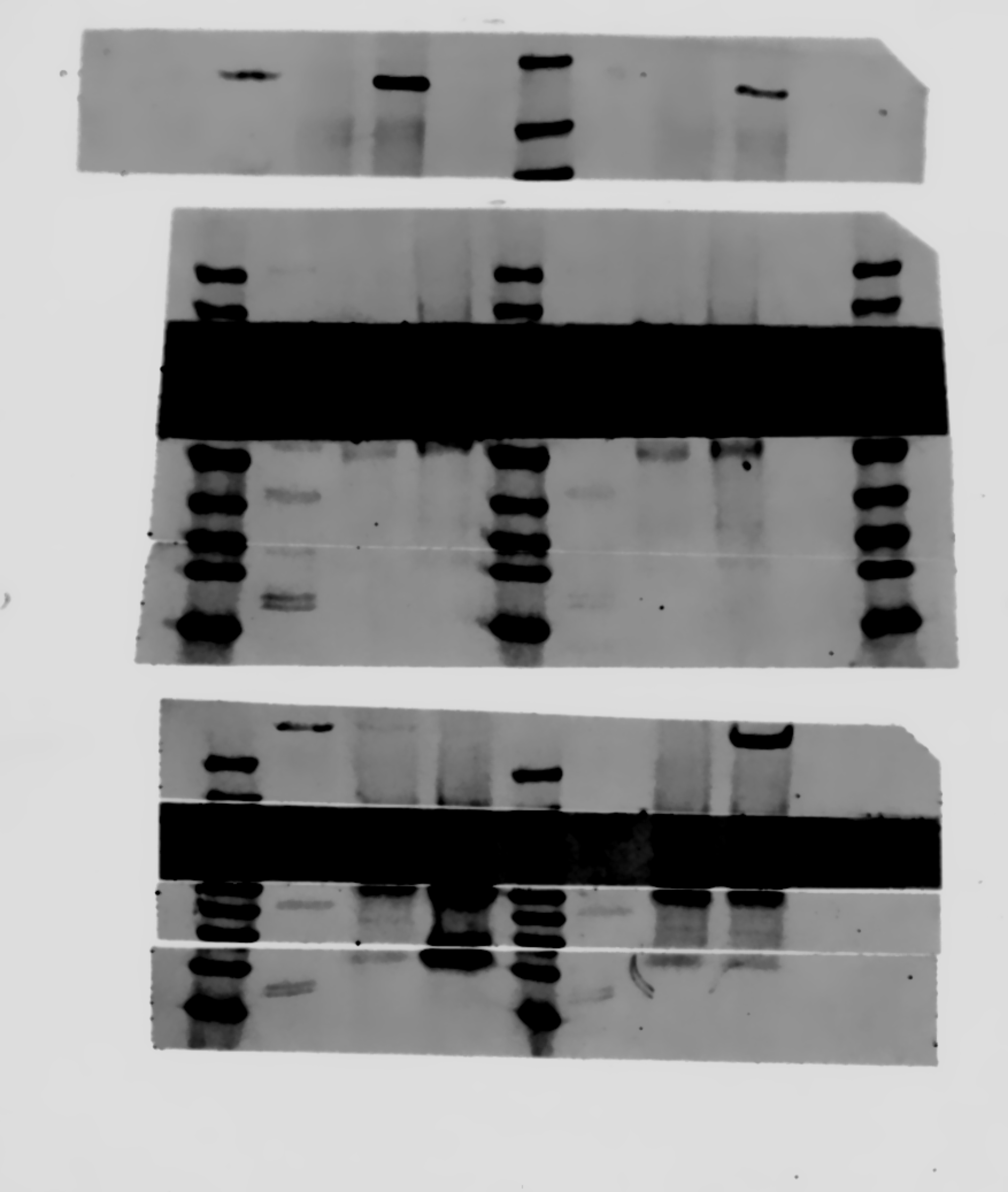

Supplement: Figure 3—figure supplement 1—source data 7. [file elife-92755-fig3-figsupp1-data7.zip › Figure 3-Figure Supplement 1-Source Data 7.tif]

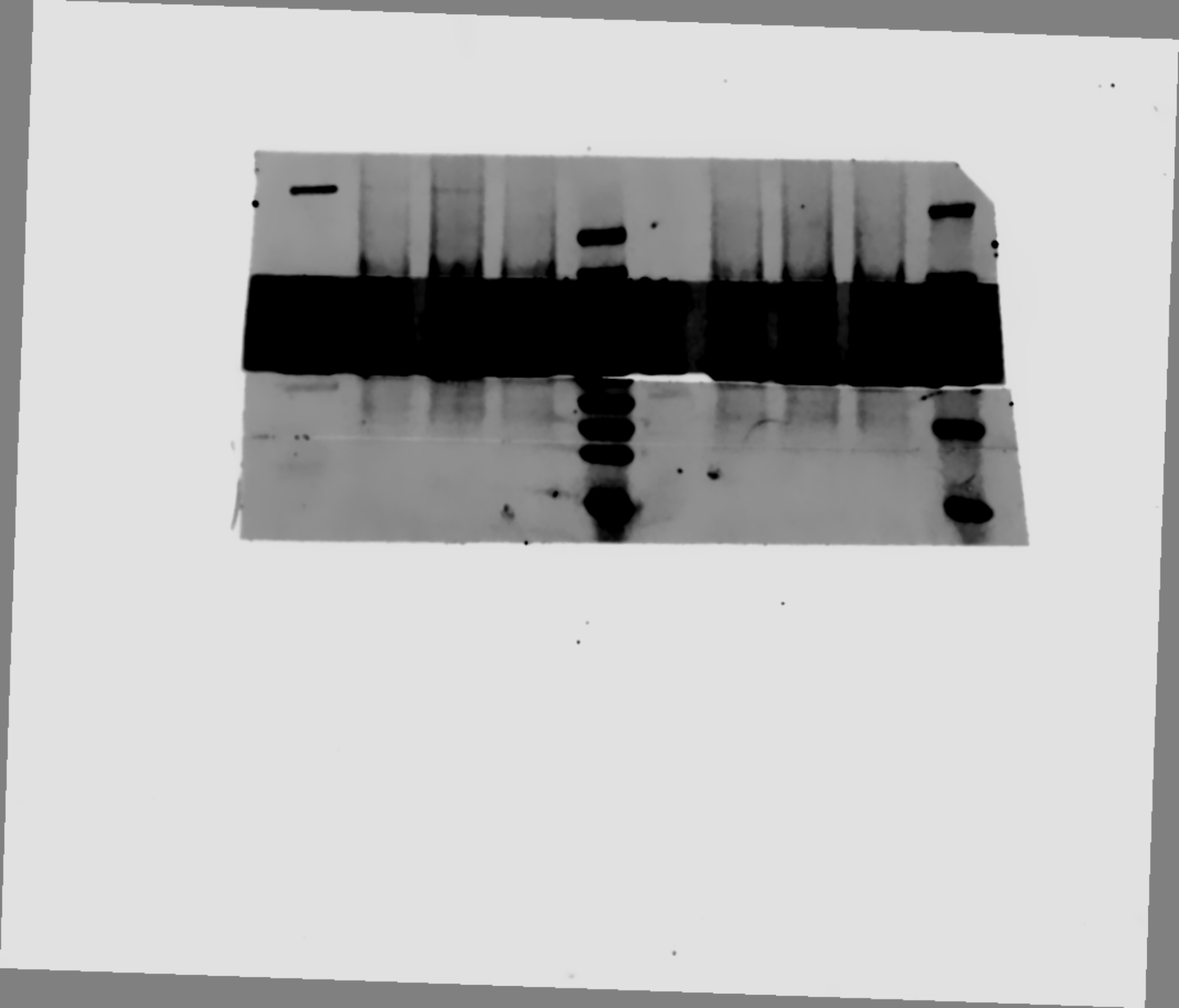

Supplement: Figure 3—figure supplement 1—source data 8. [file elife-92755-fig3-figsupp1-data8.zip › Figure 3-Figure Supplement 1-Source Data 8.tif]

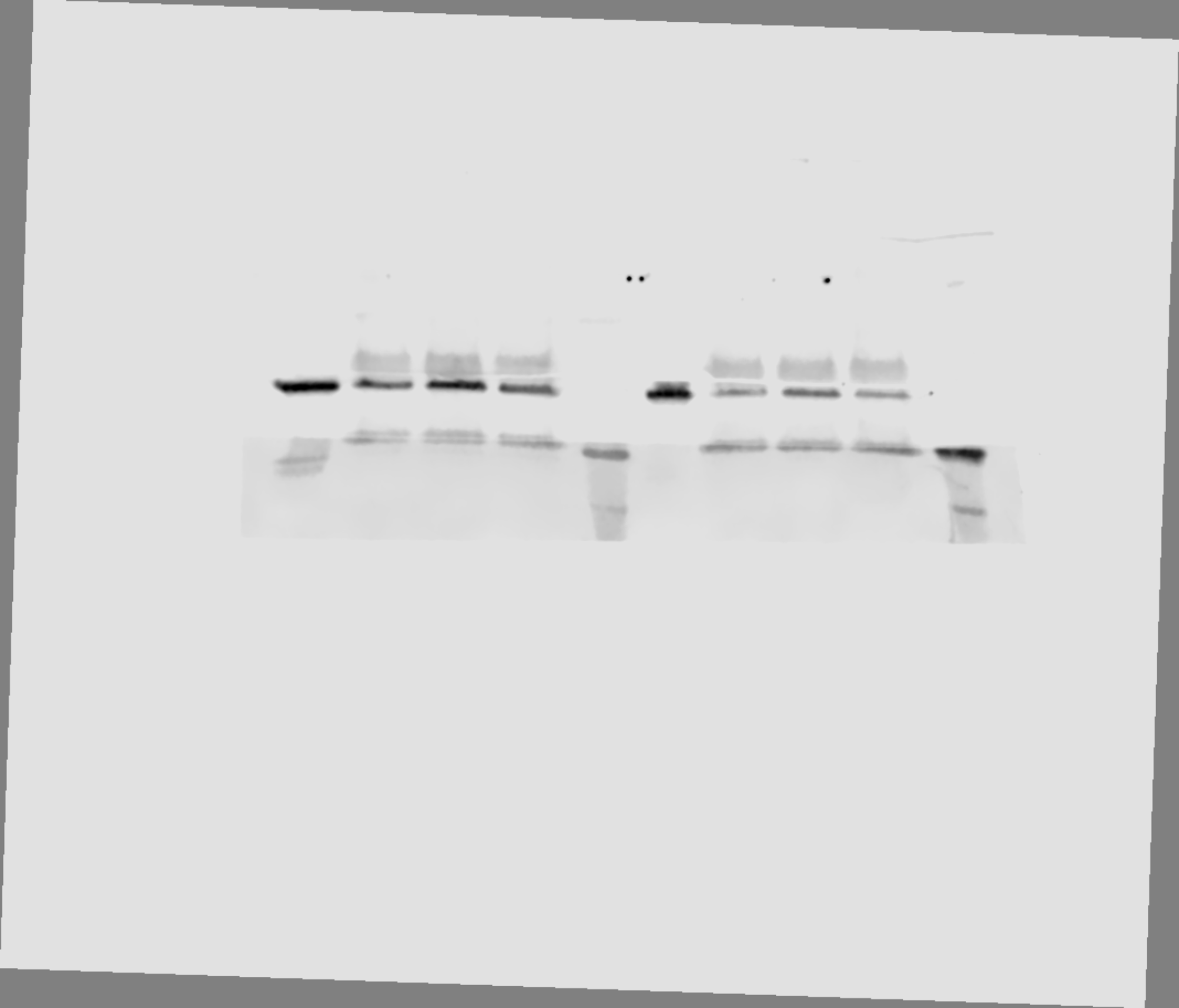

Supplement: Figure 3—figure supplement 1—source data 9. [file elife-92755-fig3-figsupp1-data9.zip › Figure 3-Figure Supplement 1-Source Data 9.tif]

Figure 3 S1

E

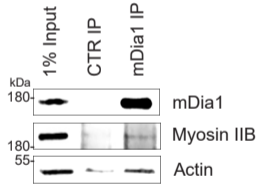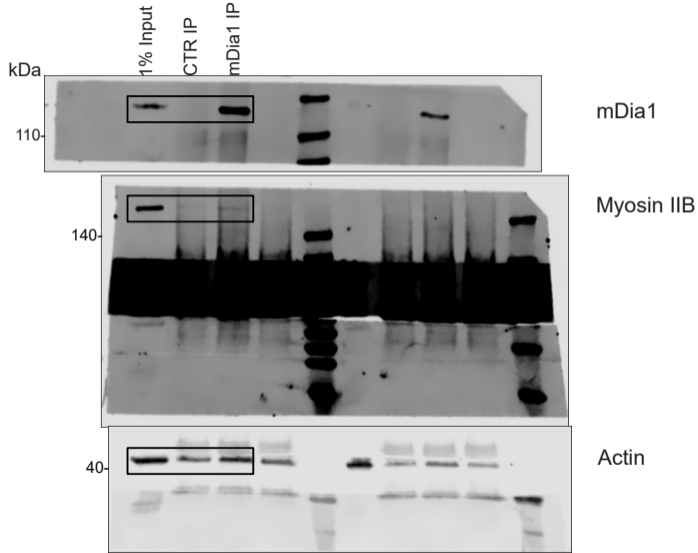

Supplement: Figure 3—figure supplement 1—source data 10. [file elife-92755-fig3-figsupp1-data10.zip › Figure 3-Figure Supplement 1-Source Data 10.pdf]

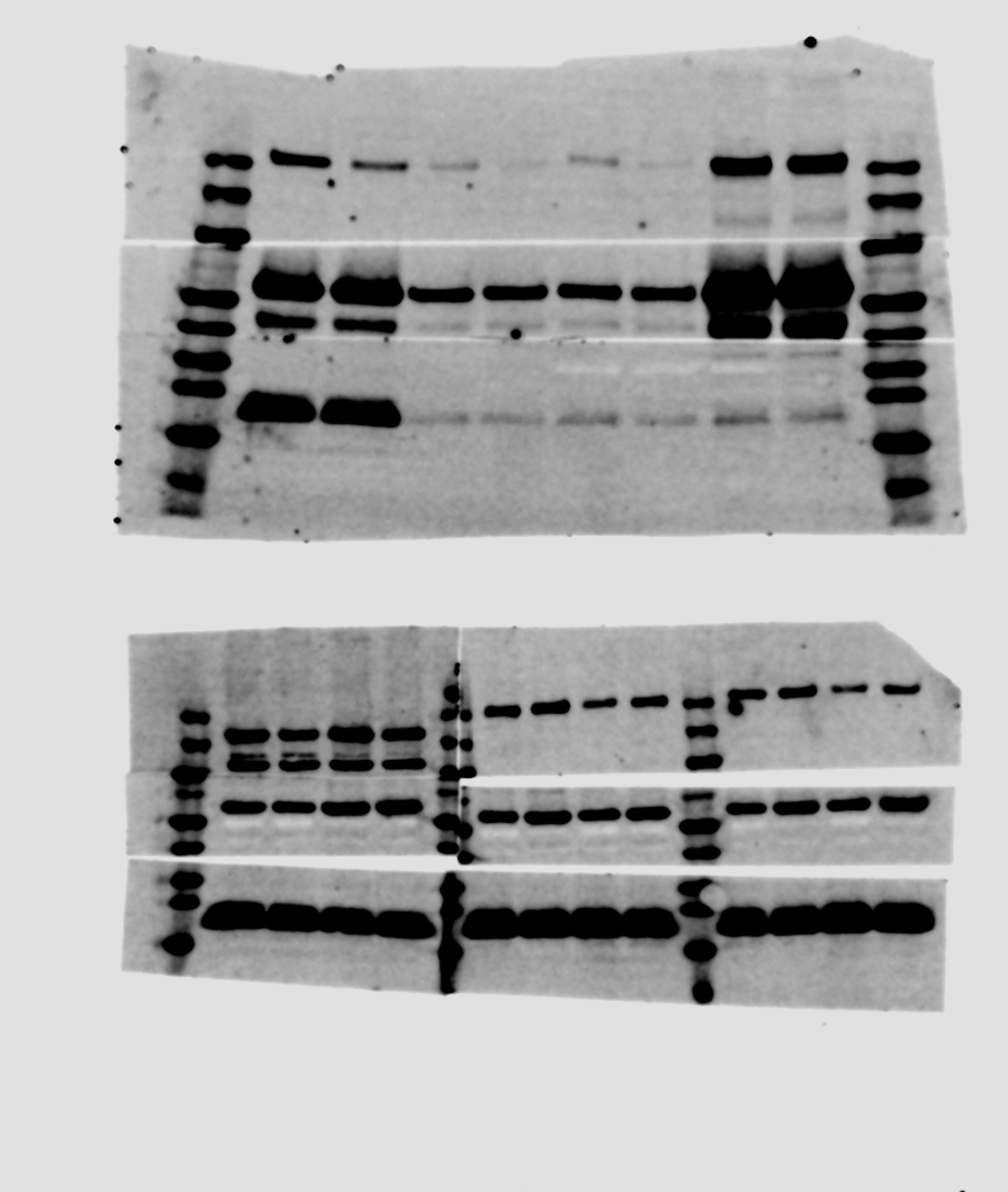

Supplement: Figure 5—source data 2. [file elife-92755-fig5-data2.zip › Figure 5-Source Data 2.tif]

Figure 5

E

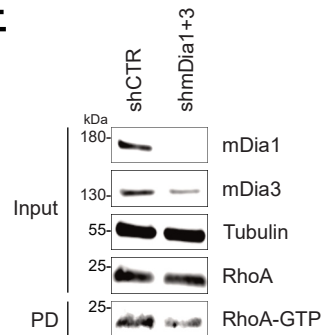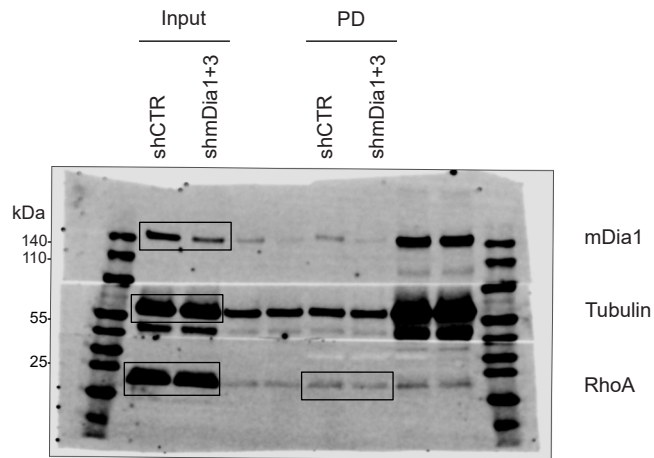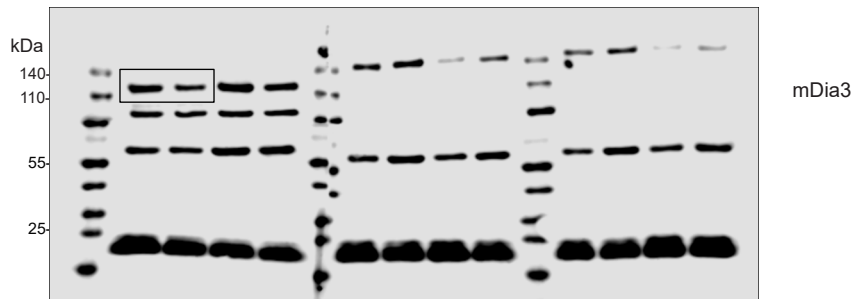

Supplement: Figure 5—source data 3. [file elife-92755-fig5-data3.zip › Figure 5-Source Data 3.pdf]

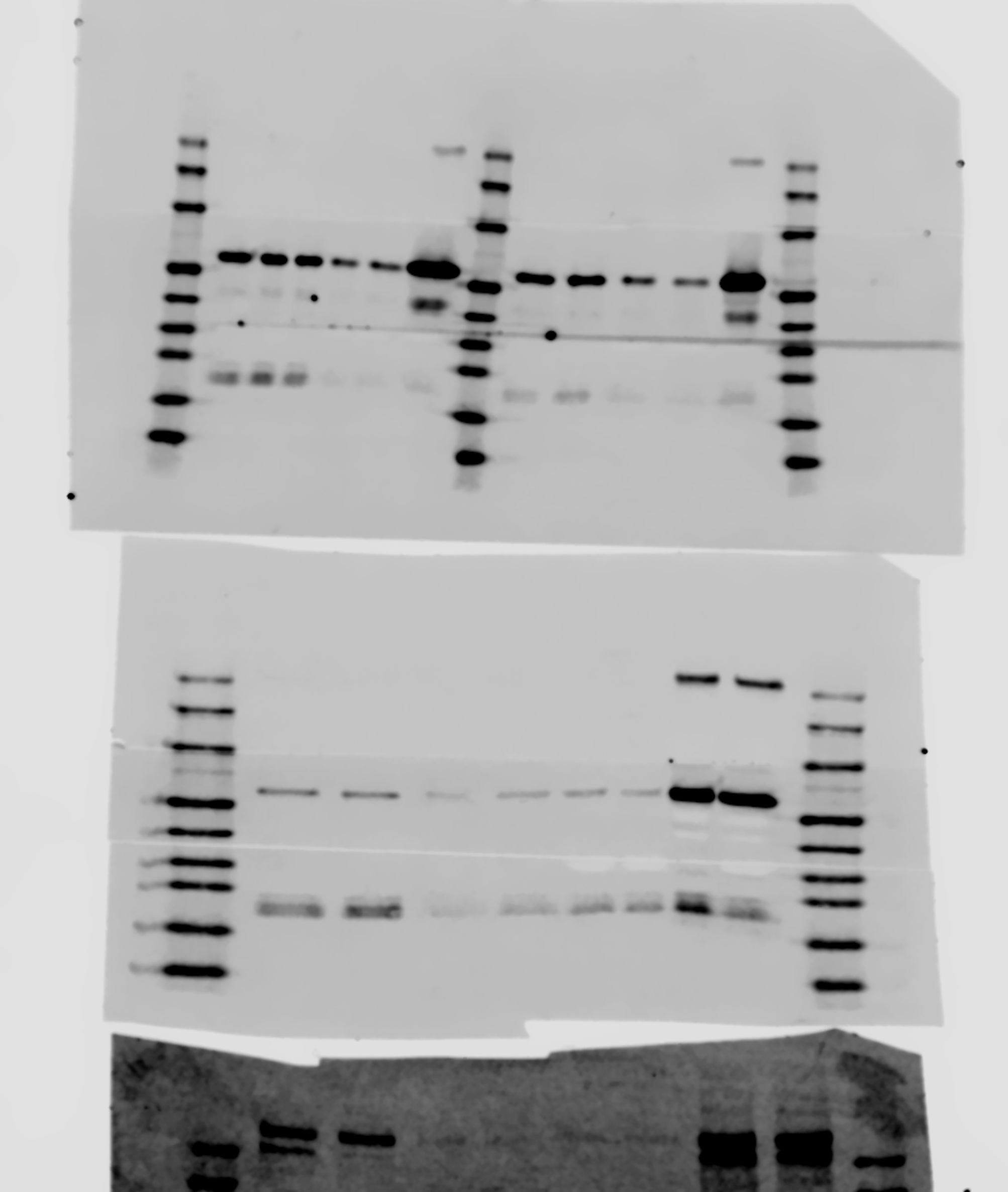

Supplement: Figure 5—source data 4. [file elife-92755-fig5-data4.zip › Figure 5-Source Data 4B.tif.tif]

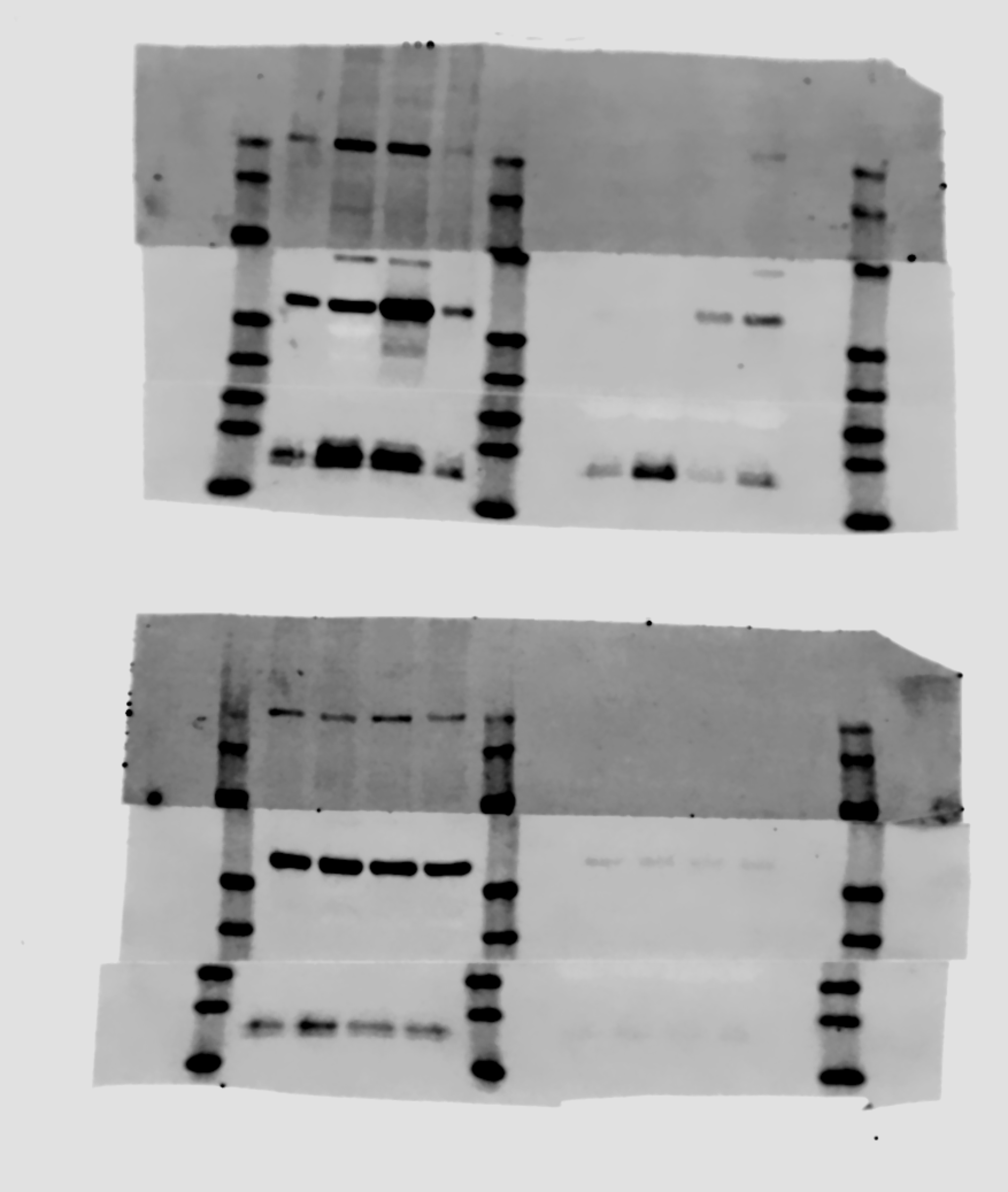

Supplement: Figure 5—source data 4. [file elife-92755-fig5-data4.zip › Figure 5-Source Data 4C.tif.tif]

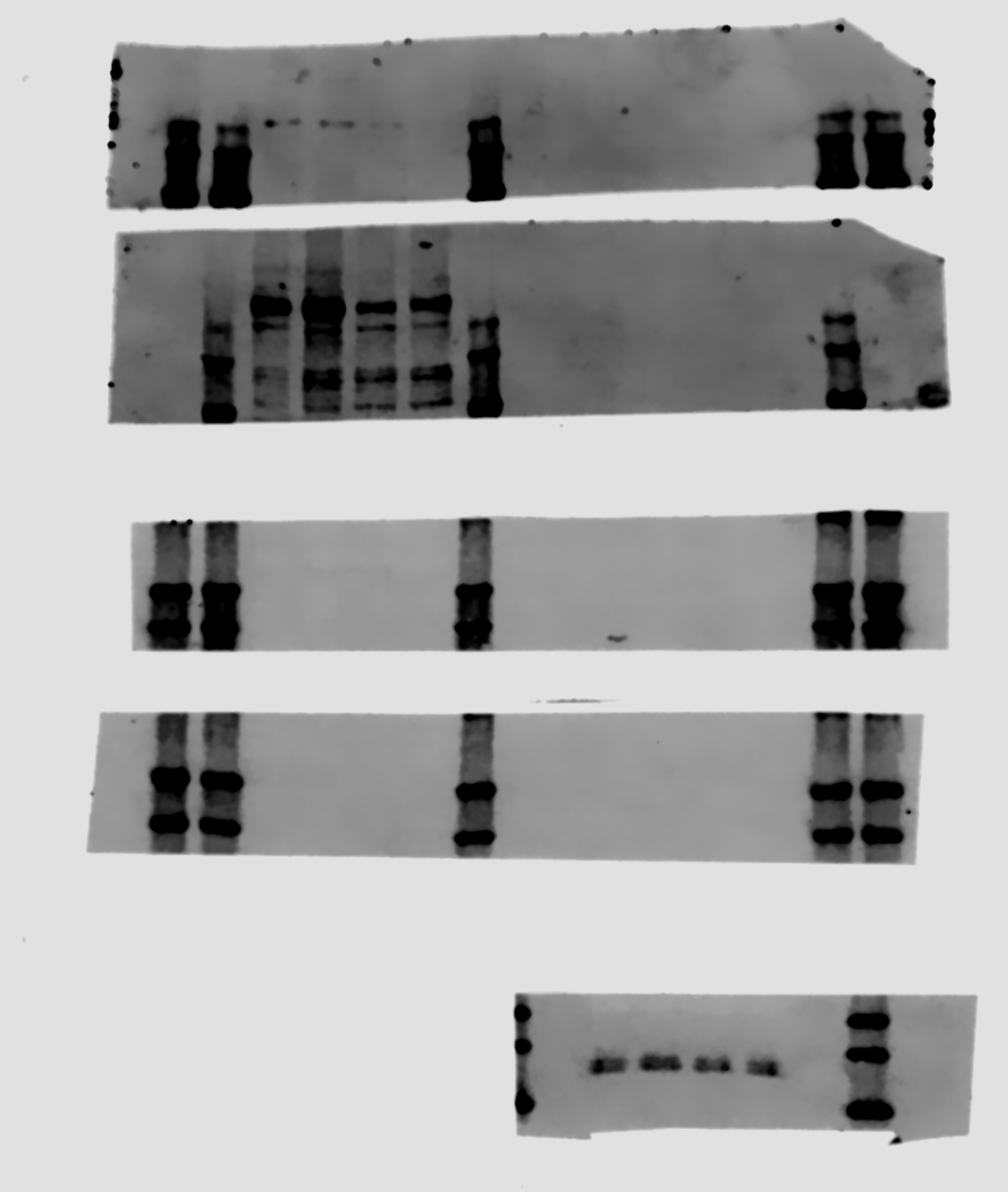

Supplement: Figure 5—source data 4. [file elife-92755-fig5-data4.zip › Figure 5-Source Data 4D.tif.tif]

# Figure 5

F

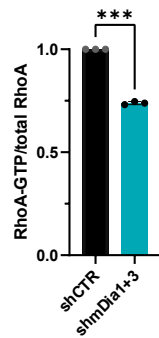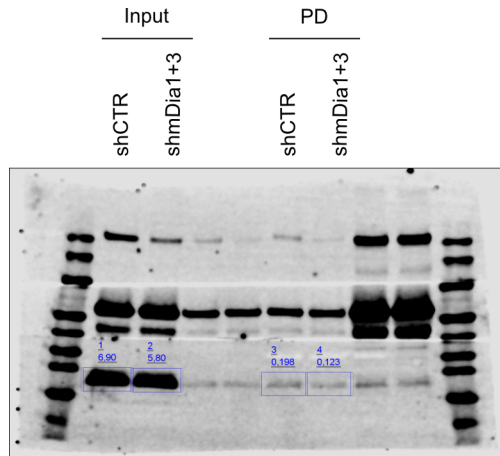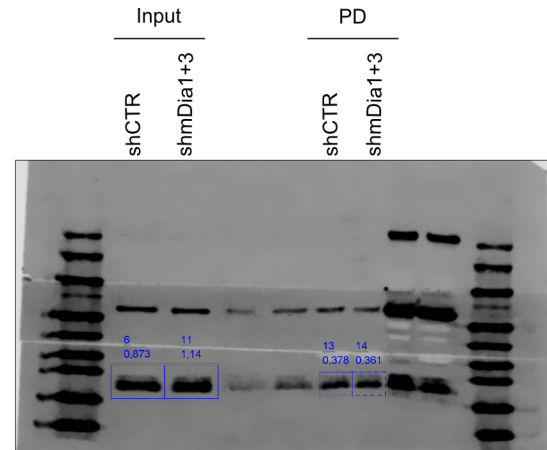

RhoA

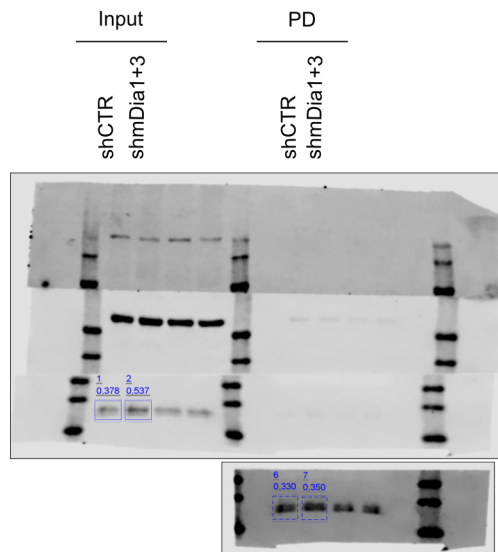

RhoA

RhoA

\* Pulldown part of blot  
reincubated with antibody  
to increase signal

Supplement: Figure 5—source data 5. [file elife-92755-fig5-data5.zip › Figure 5-Source Data 5.pdf]

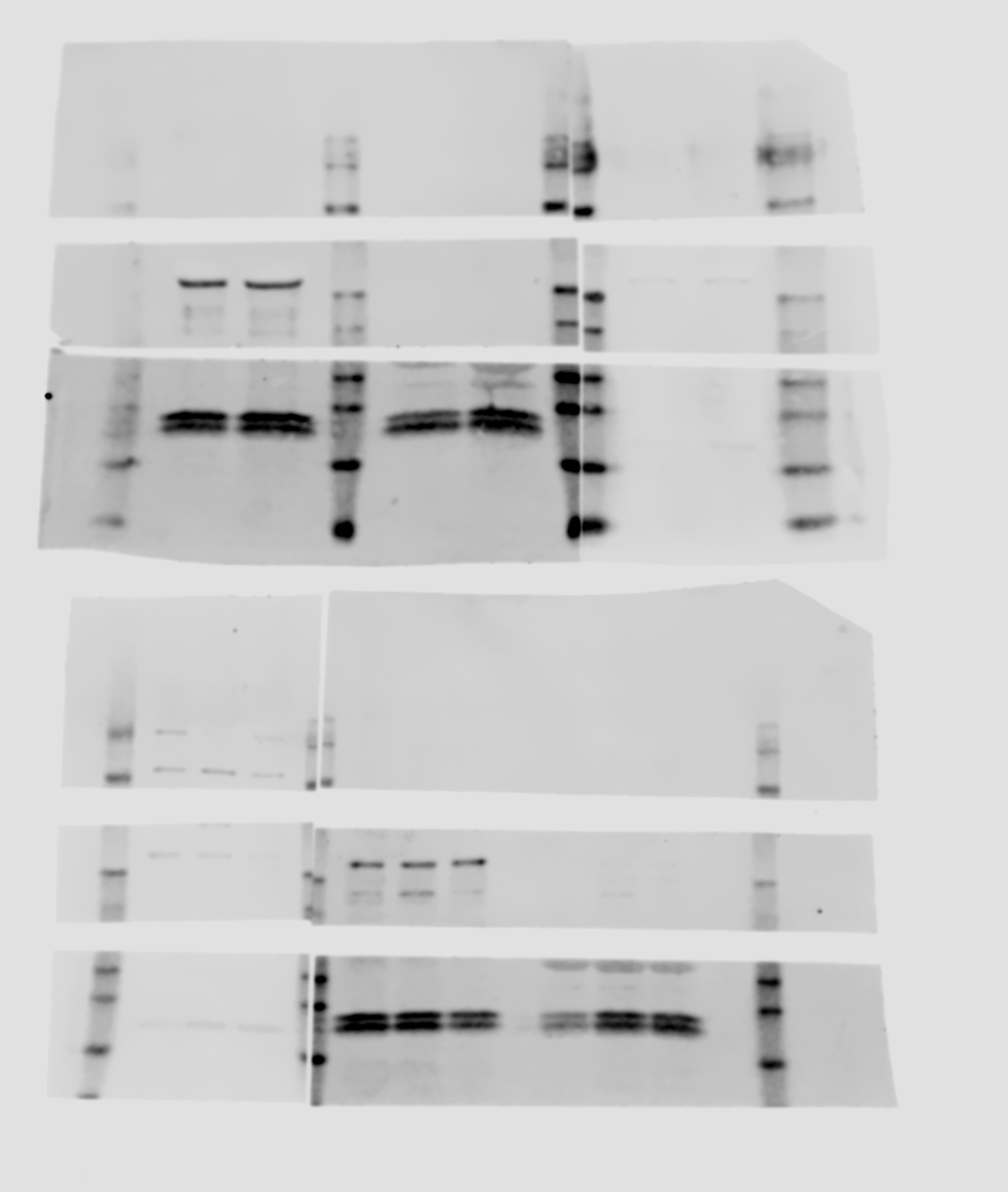

Supplement: Figure 6—source data 1. [file elife-92755-fig6-data1.zip › Figure 6-Source Data 1.tif]

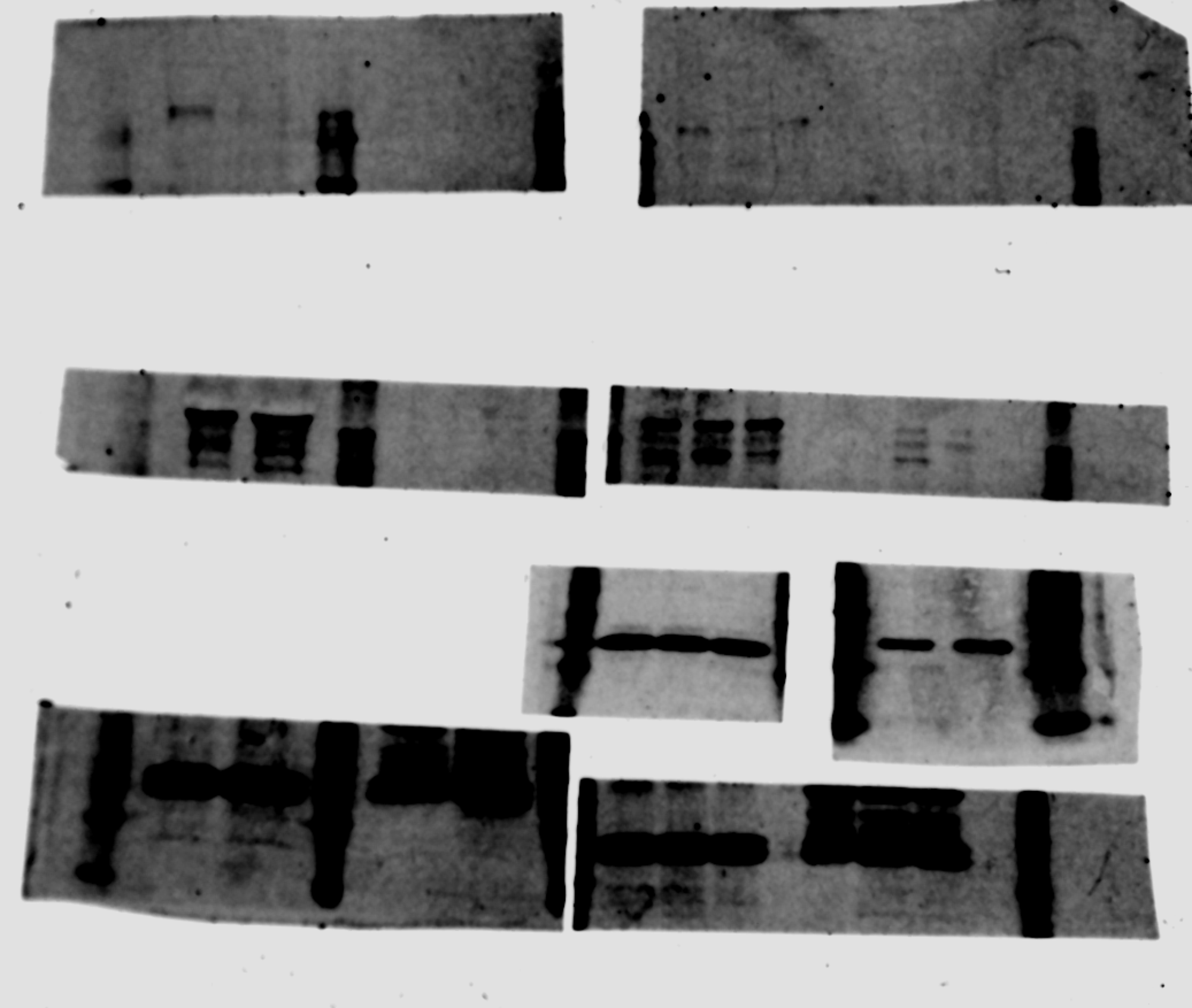

Supplement: Figure 6—source data 2. [file elife-92755-fig6-data2.zip › Figure 6-Source Data 2.tif]

Figure 6

B

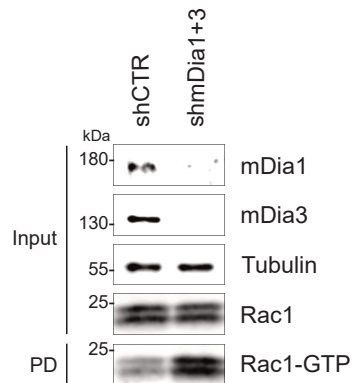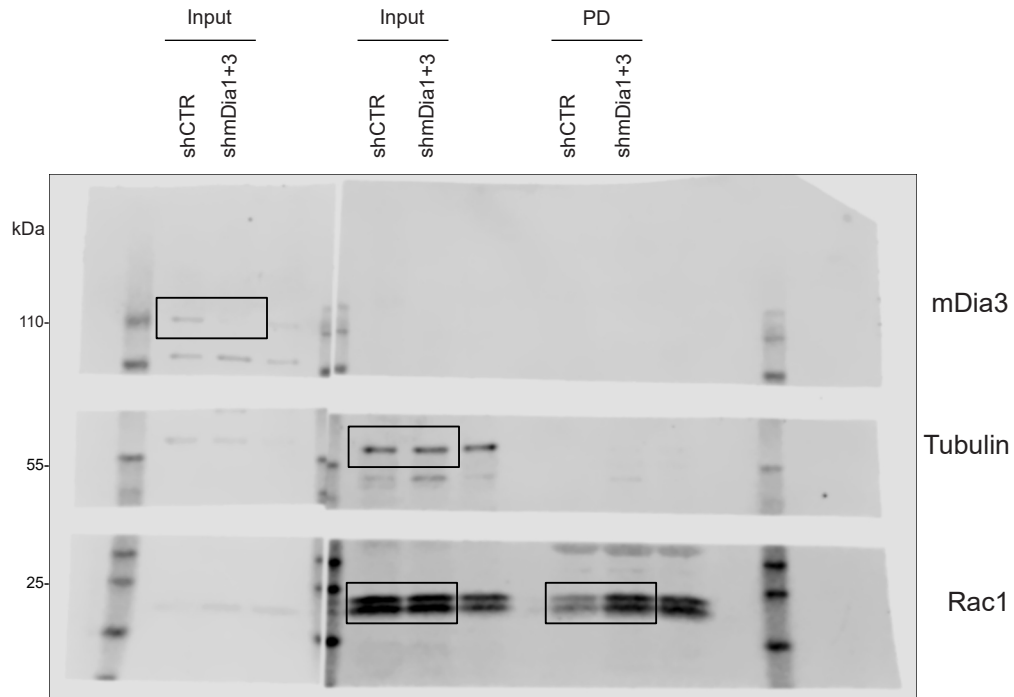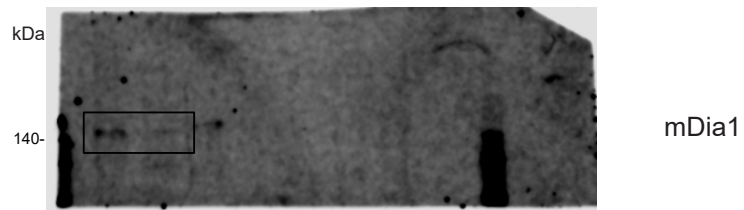

Supplement: Figure 6—source data 3. [file elife-92755-fig6-data3.zip › Figure 6-Source Data 3.pdf]

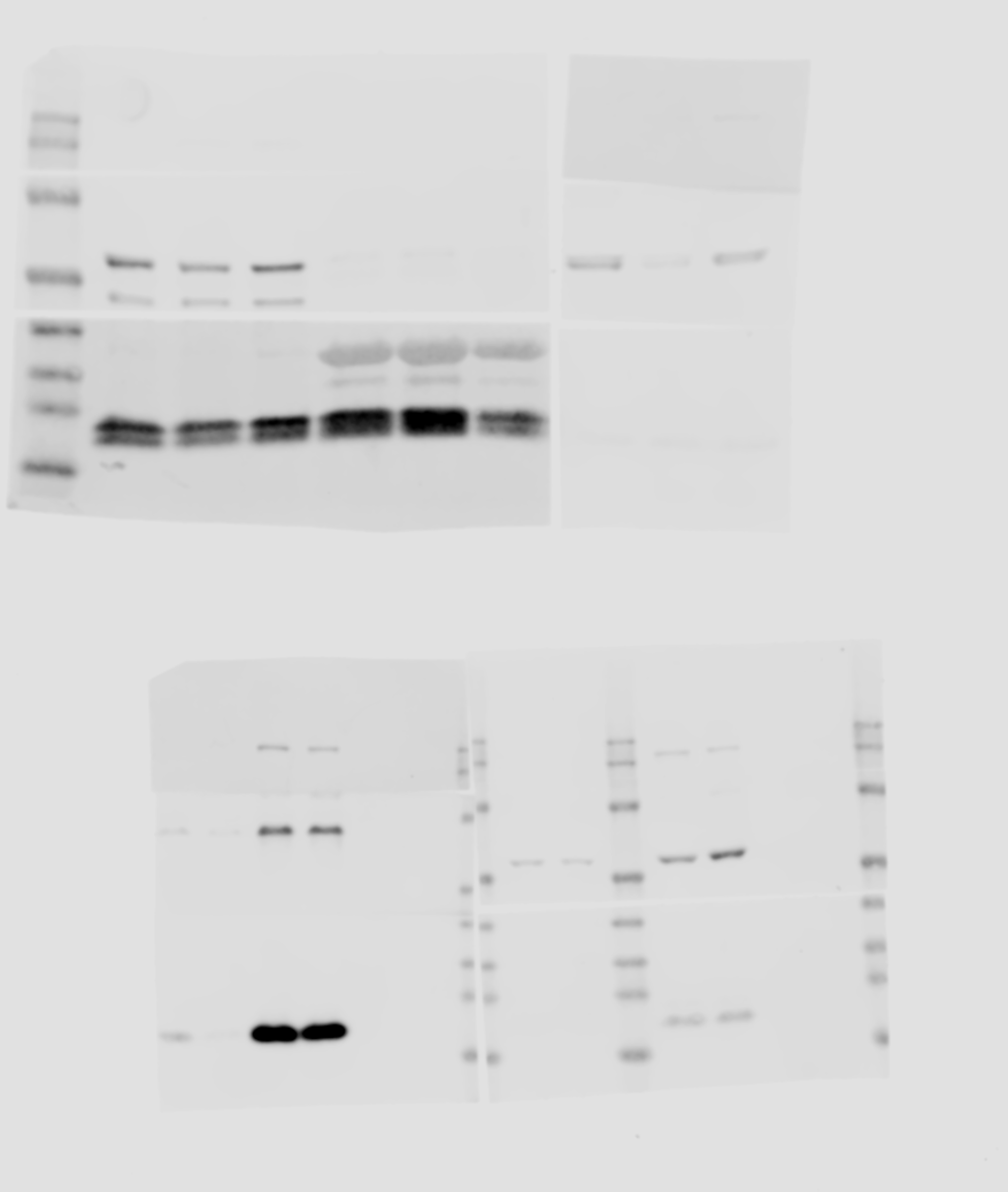

Supplement: Figure 6—source data 5. [file elife-92755-fig6-data5.zip › Figure 6-Source Data 5B.tif]

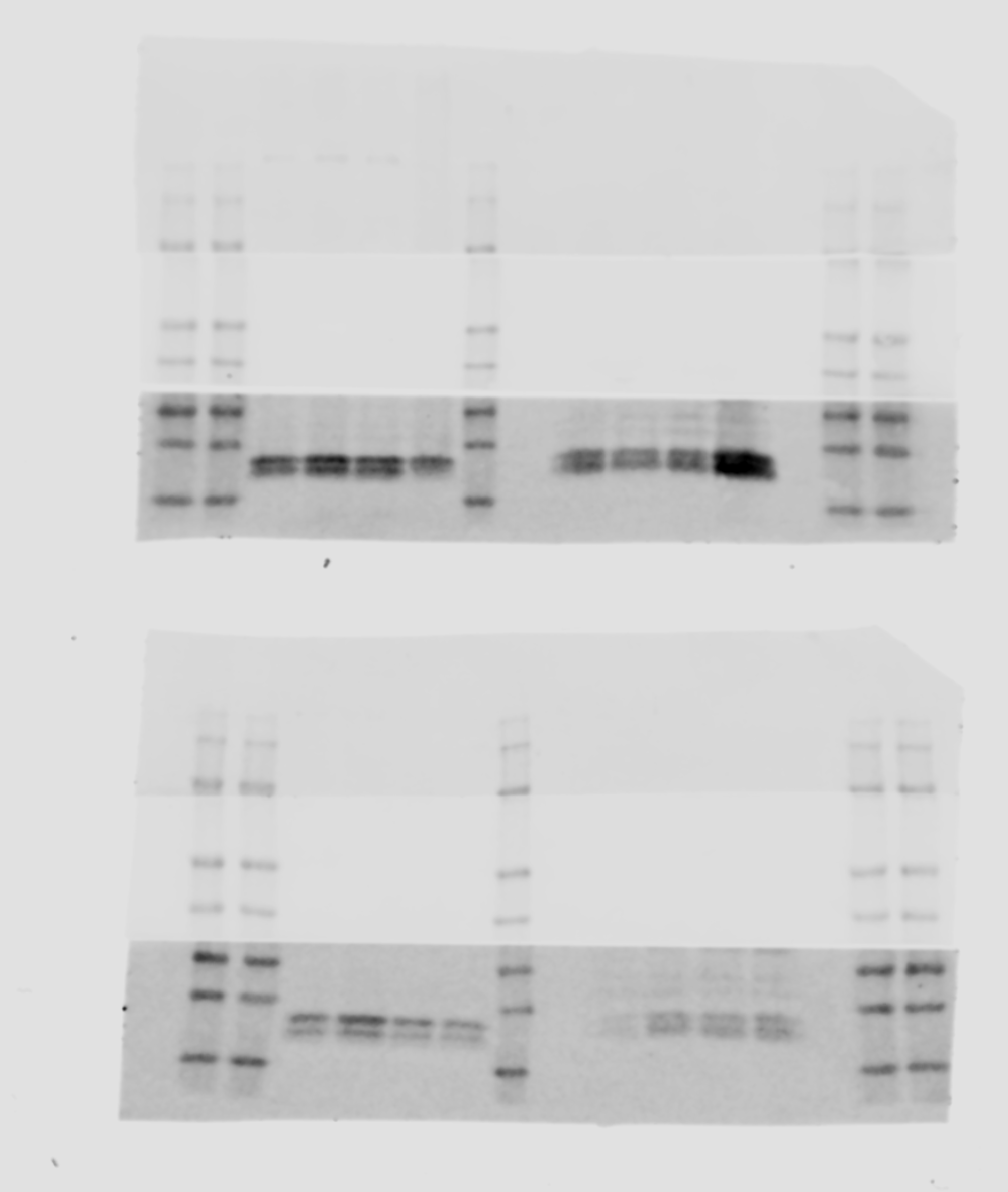

Supplement: Figure 6—source data 5. [file elife-92755-fig6-data5.zip › Figure 6-Source Data 5C.tif]

Figure 6

C

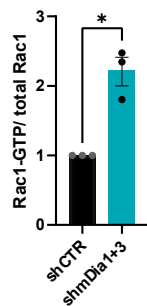

Input PD

shCTR shmDia1+3 shCTR shmDia1+3

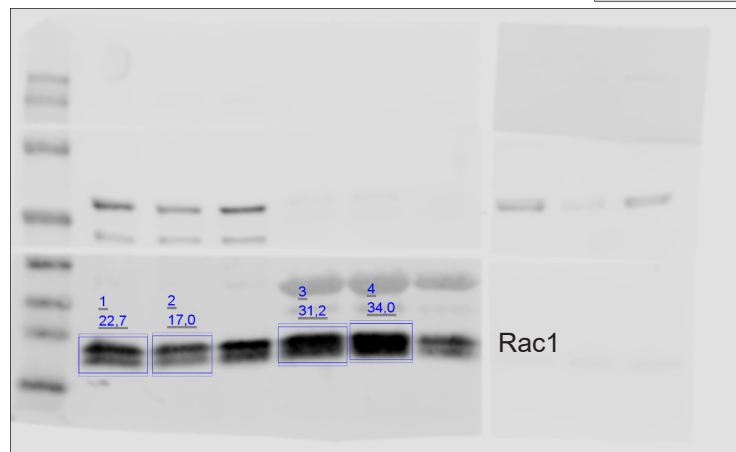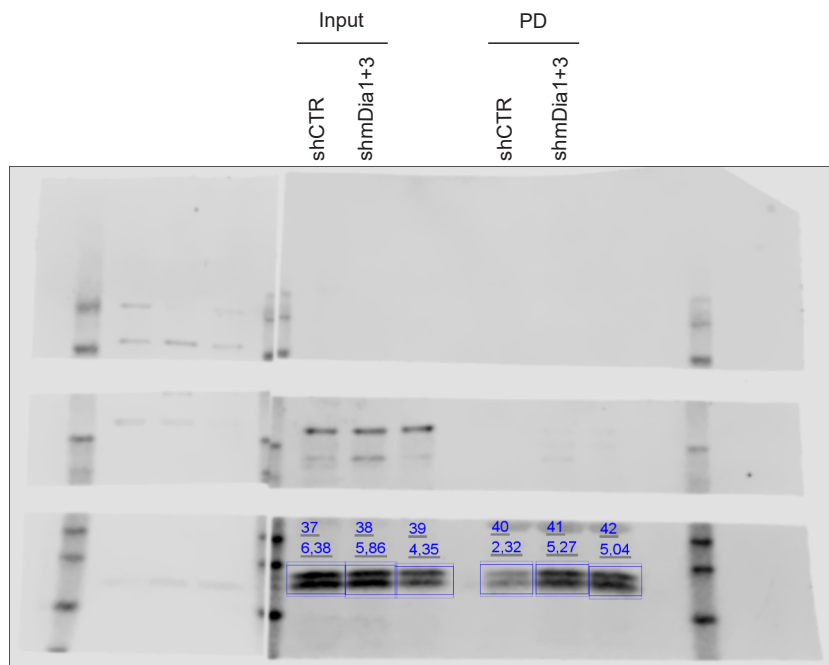

Rac1

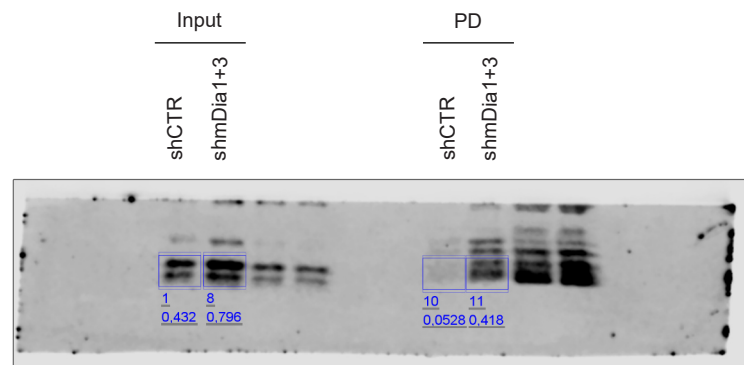

Rac1

Supplement: Figure 6—source data 6. [file elife-92755-fig6-data6.zip › Figure 6-Source Data 6.pdf]

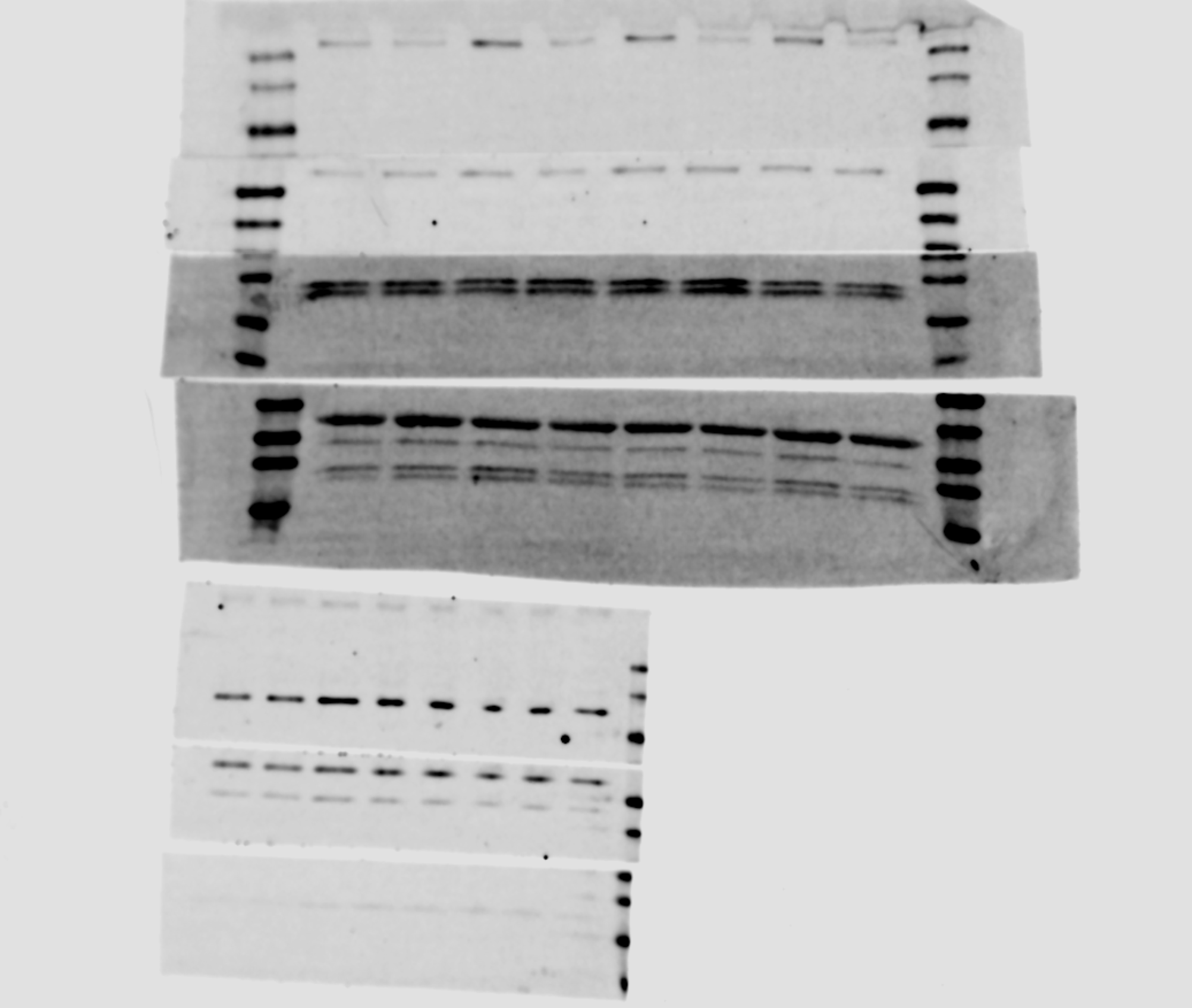

Supplement: Figure 6—figure supplement 1—source data 1. [file elife-92755-fig6-figsupp1-data1.zip › Figure 6-Figure Supplement 1-Source Data 1.tif]

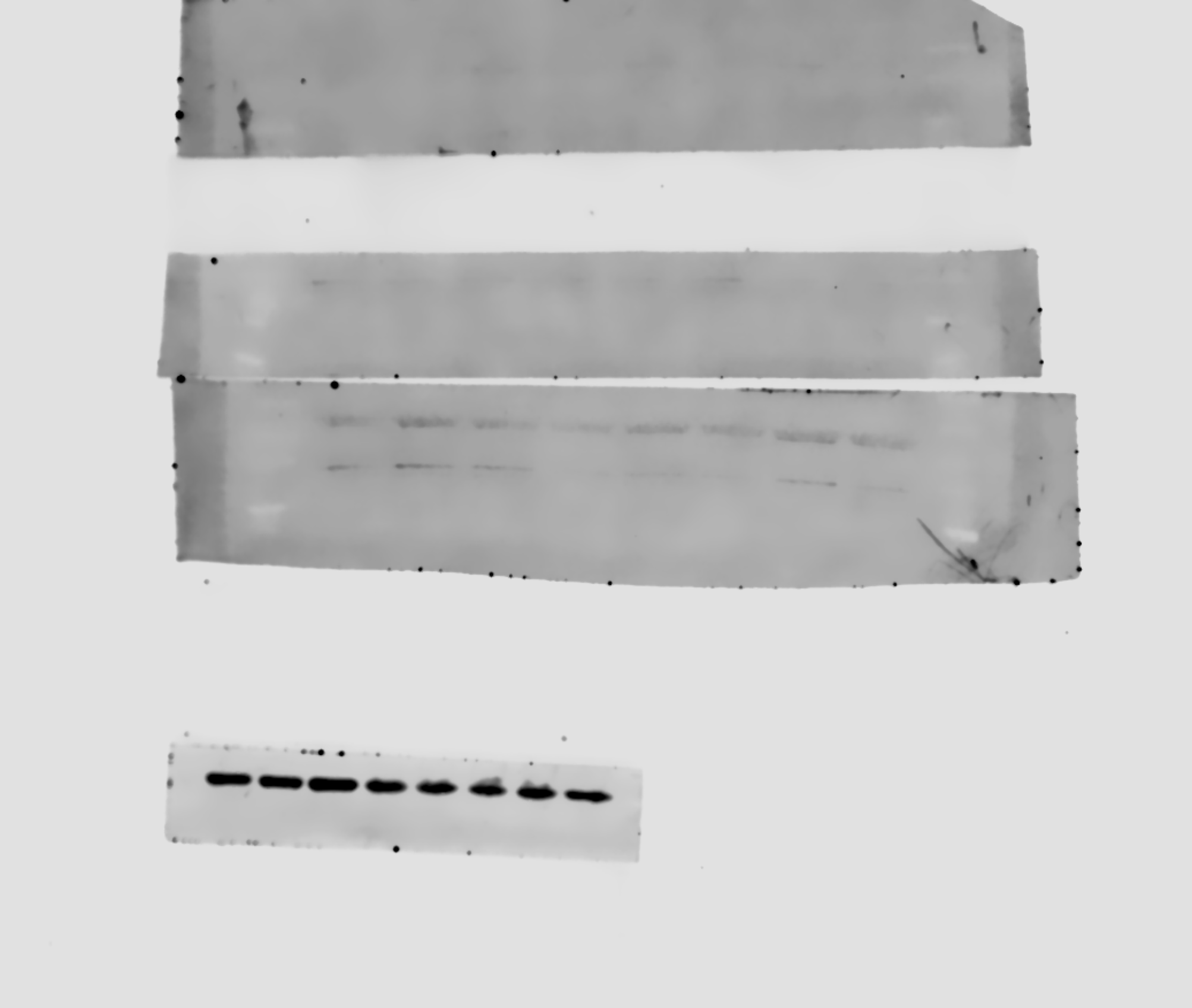

Supplement: Figure 6—figure supplement 1—source data 2. [file elife-92755-fig6-figsupp1-data2.zip › Figure 6-Figure Supplement 1-Source Data 2.tif]

Figure 6 S1

A

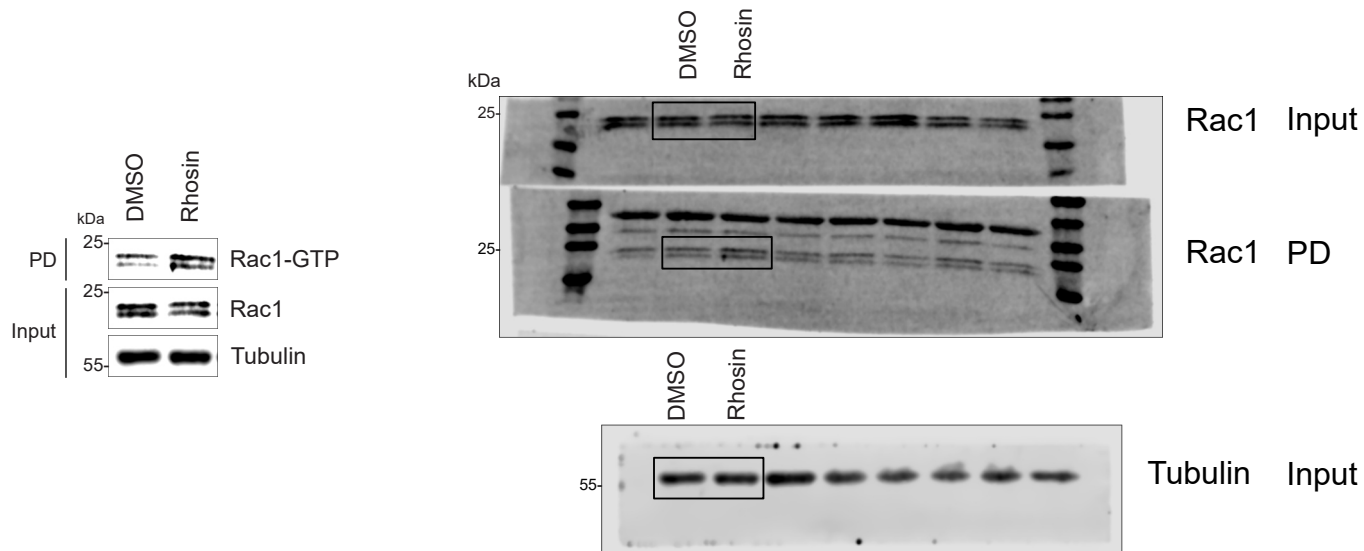

Supplement: Figure 6—figure supplement 1—source data 3. [file elife-92755-fig6-figsupp1-data3.zip › Figure 6-Figure Supplement 1-Source Data 3.pdf]

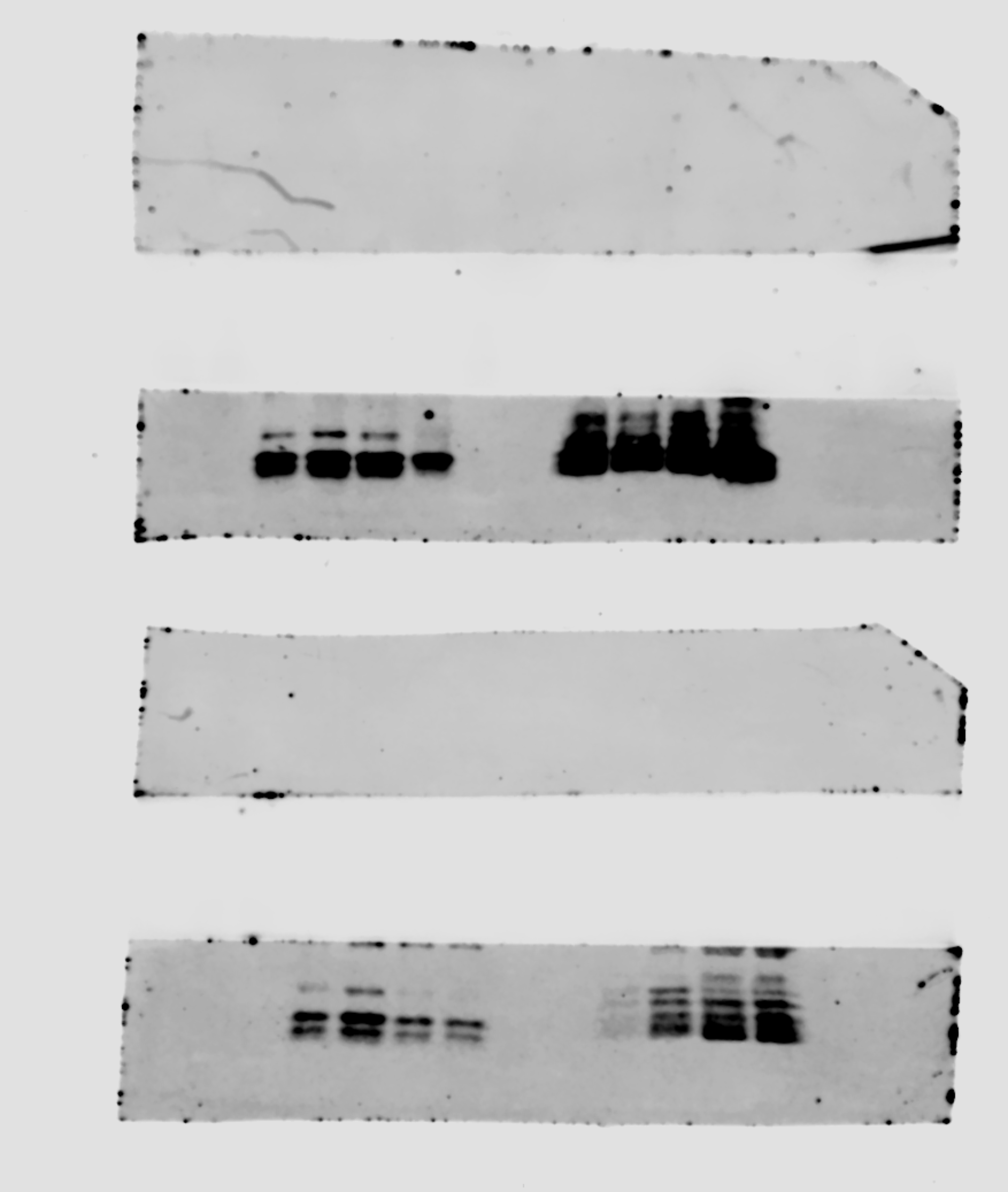

Supplement: Figure 6—figure supplement 1—source data 5. [file elife-92755-fig6-figsupp1-data5.zip › Figure 6-Figure Supplement 1-Source Data 5A.tif]

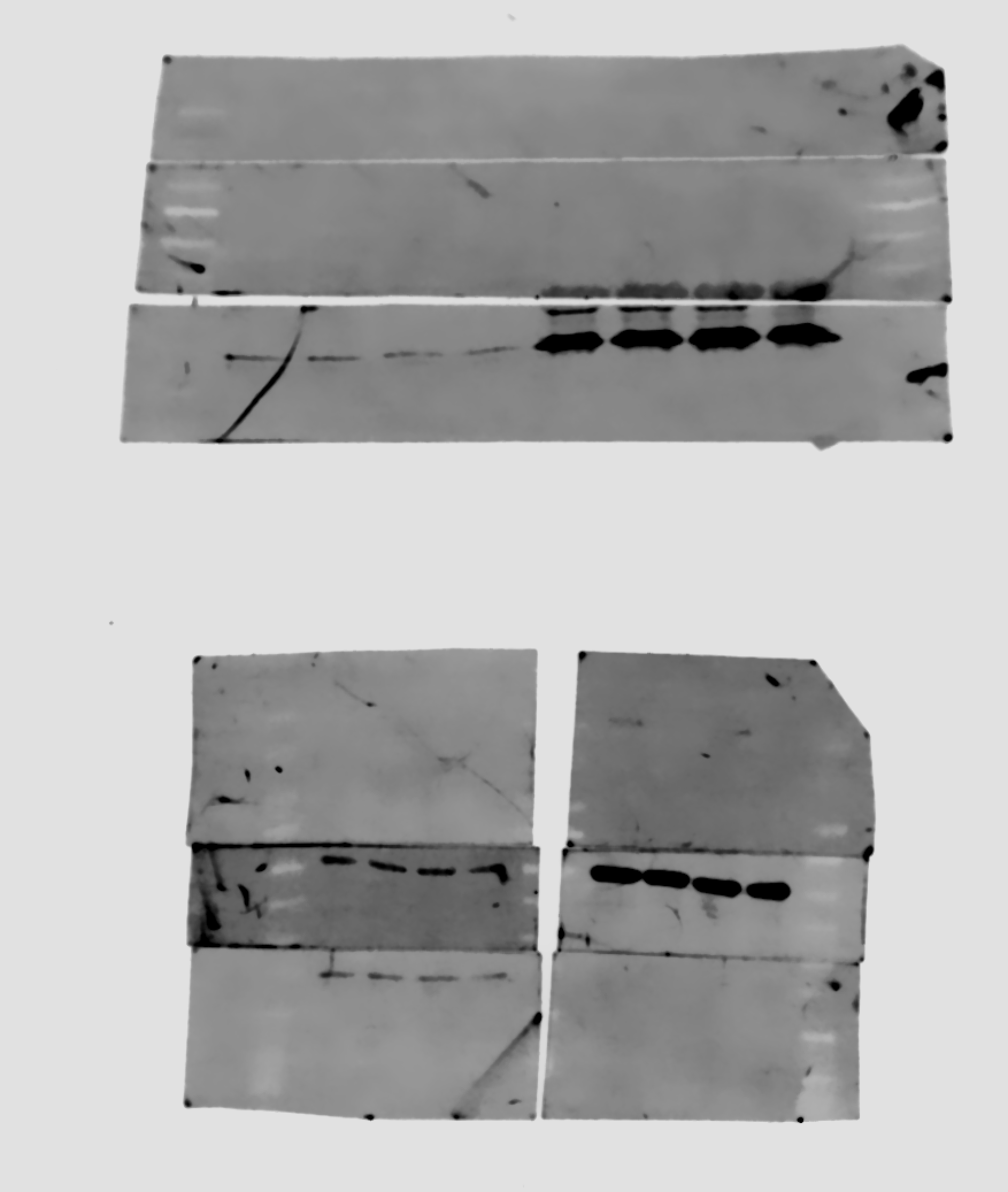

Supplement: Figure 6—figure supplement 1—source data 5. [file elife-92755-fig6-figsupp1-data5.zip › Figure 6-Figure Supplement 1-Source Data 5B.tif]

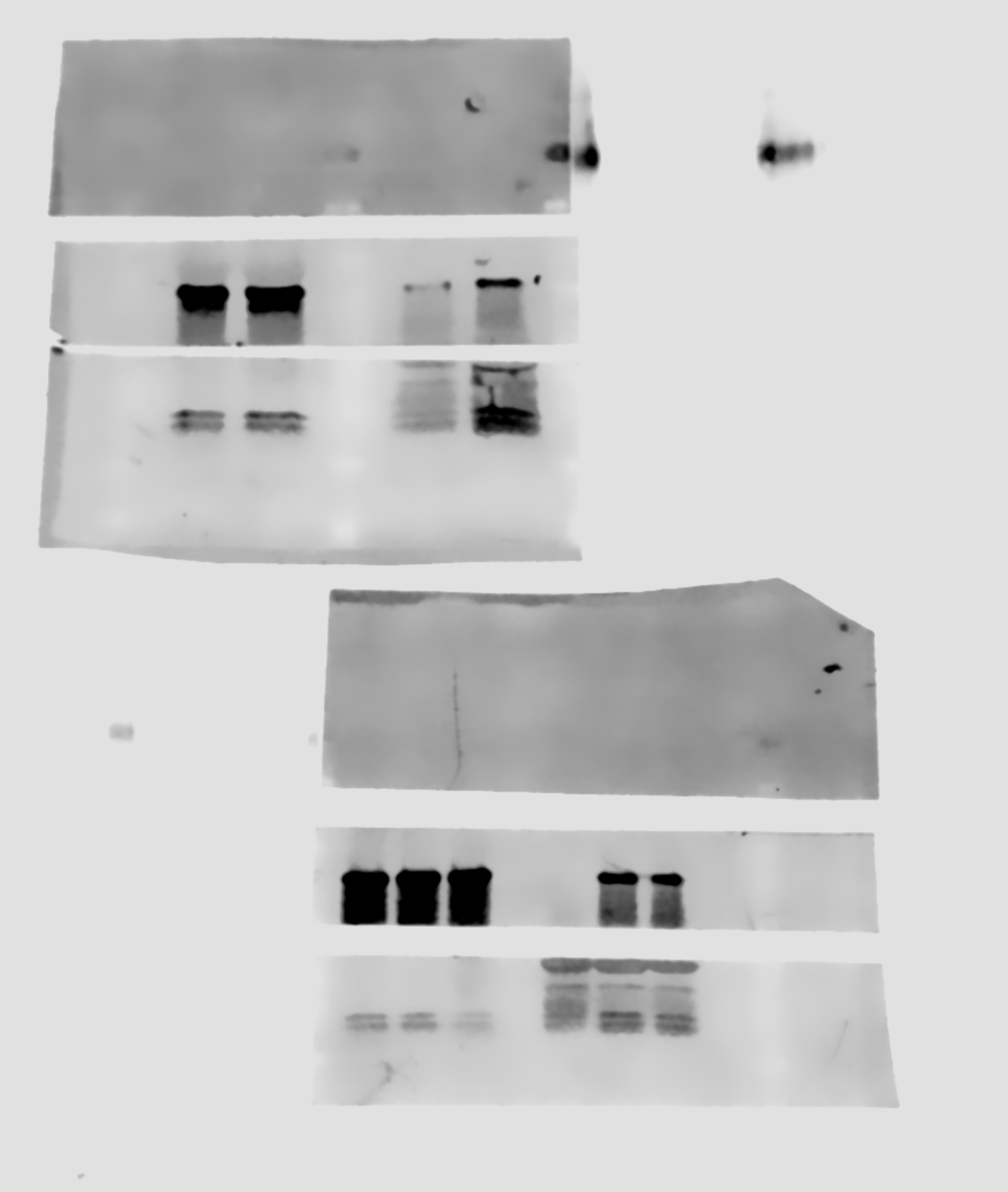

Supplement: Figure 6—figure supplement 1—source data 5. [file elife-92755-fig6-figsupp1-data5.zip › Figure 6-Figure Supplement 1-Source Data 5C.tif]

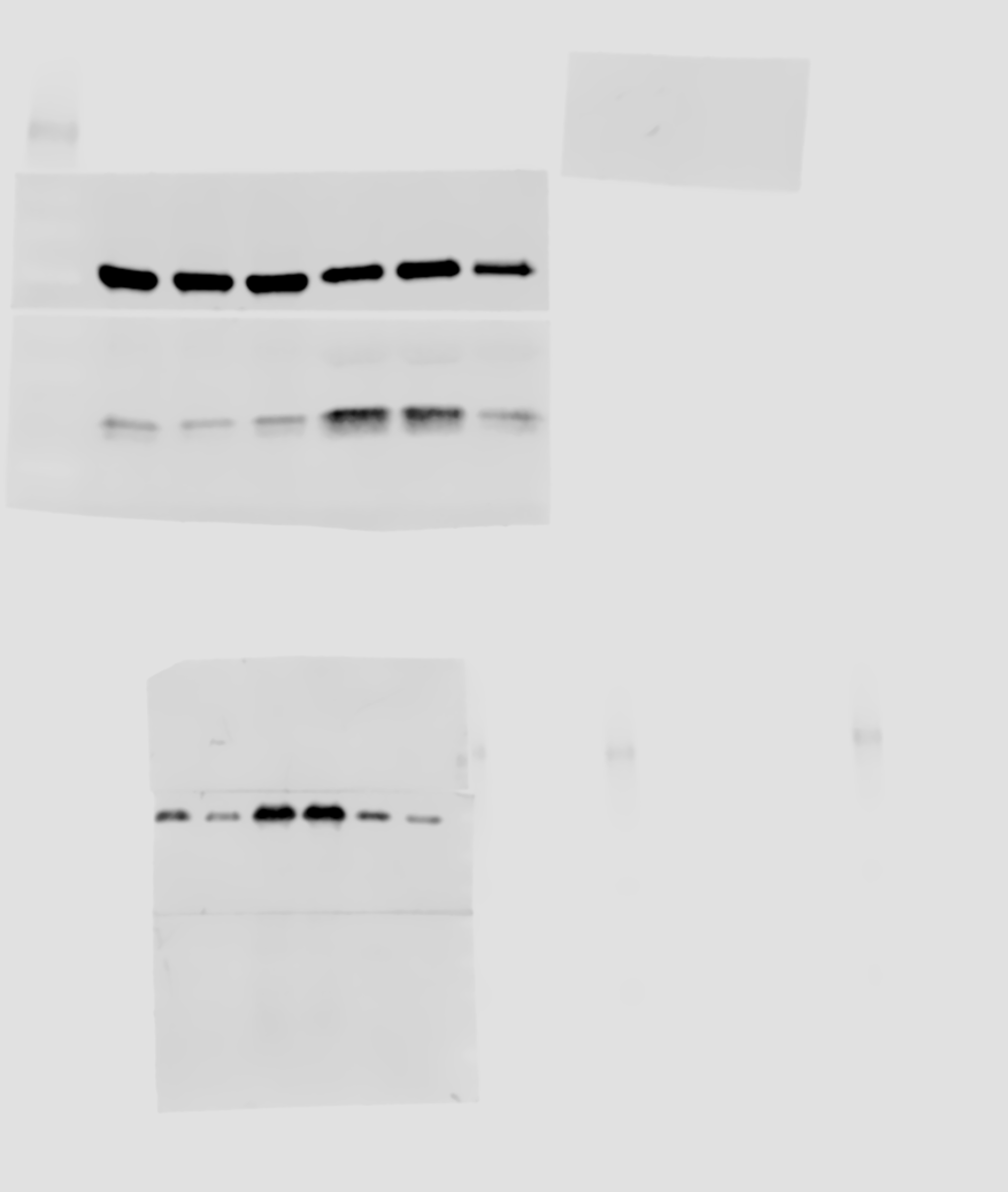

Supplement: Figure 6—figure supplement 1—source data 5. [file elife-92755-fig6-figsupp1-data5.zip › Figure 6-Figure Supplement 1-Source Data 5D.tif]

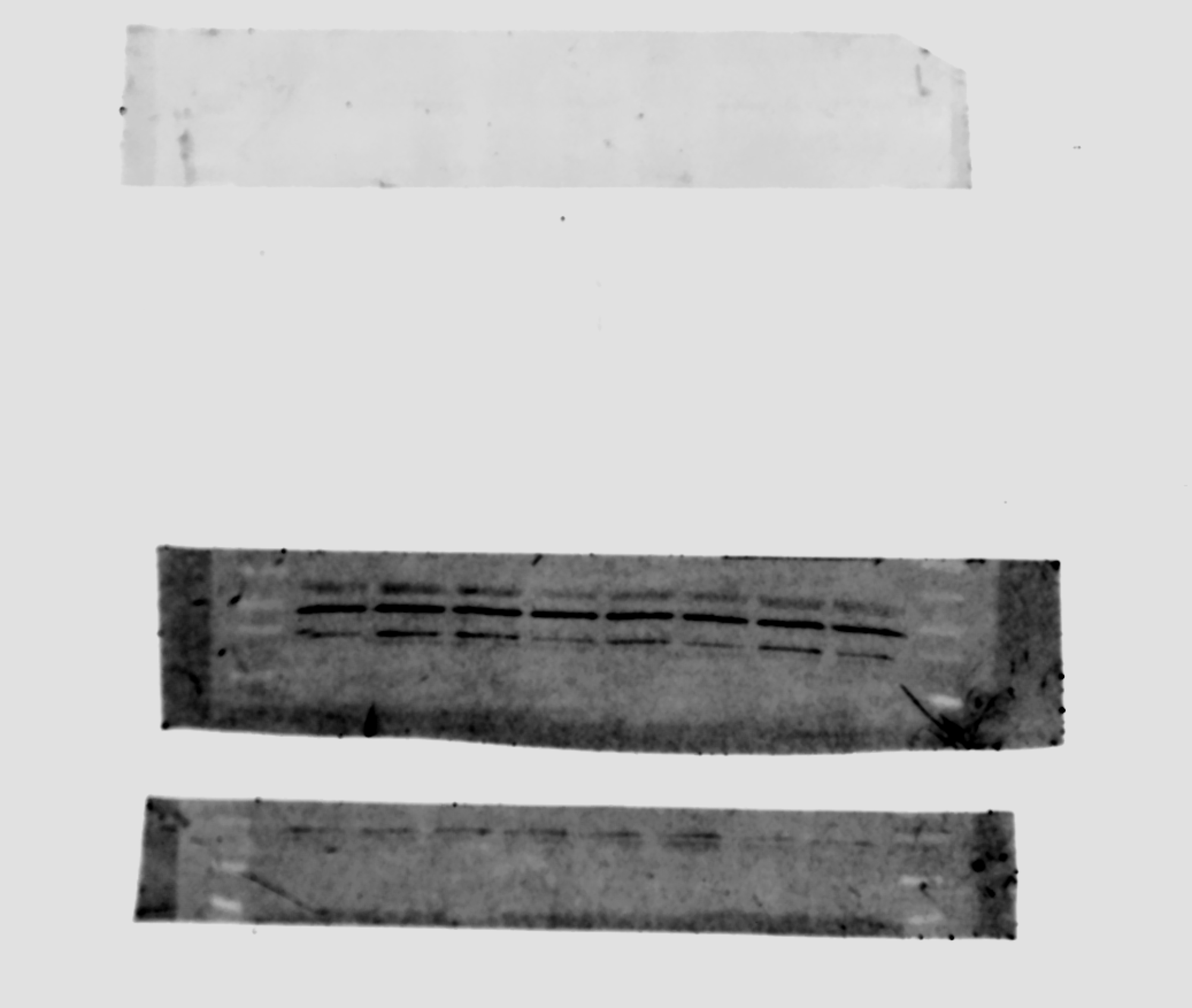

Supplement: Figure 6—figure supplement 1—source data 5. [file elife-92755-fig6-figsupp1-data5.zip › Figure 6-Figure Supplement 1-Source Data 5E.tif]

Figure 6 S1

G

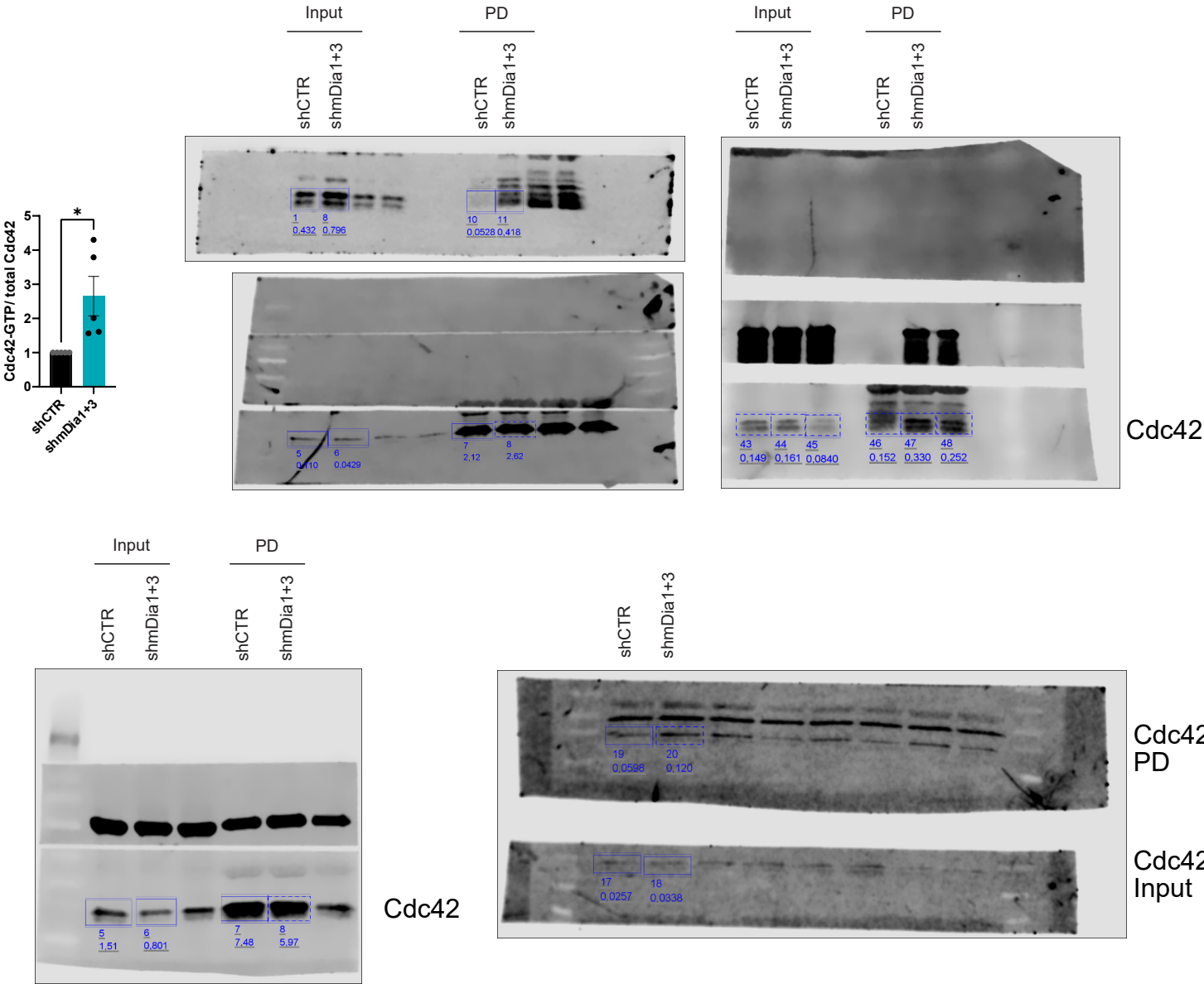

Supplement: Figure 6—figure supplement 1—source data 6. [file elife-92755-fig6-figsupp1-data6.zip › Figure 6-Figure Supplement 1-Source Data 6.pdf]
