# Supplementary material for: Rho GTPase signaling and mDia facilitate endocytosis via presynaptic actin
Source: eLife. 2024 Mar 19;12:RP92755. doi: 10.7554/eLife.92755 (PMC10950329; doi:10.7554/eLife.92755)
Supplement: Figure 3—figure supplement 1—source data 6. [file elife-92755-fig3-figsupp1-data6.zip › Figure 3-Figure Supplement 1-Source Data 6.pdf]

Figure 3 S1

B

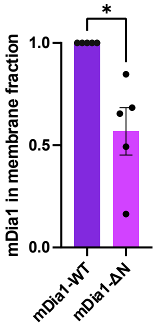

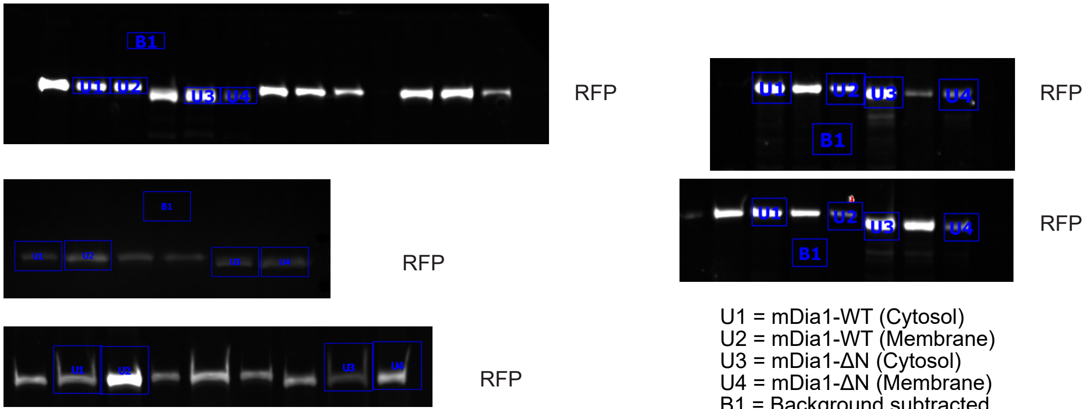

U1 = mDia1-WT (Cytosol)
U2 = mDia1-WT (Membrane)
U3 = mDia1-ΔN (Cytosol)
U4 = mDia1-ΔN (Membrane)
B1 = Background subtracted
